# Identification of a basement membrane-related genes signature with immune correlation in bladder urothelial carcinoma and verification in vitro

**DOI:** 10.1186/s12885-023-11340-0

**Published:** 2023-10-23

**Authors:** Yanze Li, Kai Xu, Ye Zhang, Hu Mao, Qiangmin Qiu, Zhiwei Yan, Xiuheng Liu, Yang Du, Zhiyuan Chen

**Affiliations:** 1 Department of Urology, Renmin Hospital, Wuhan University, Wuhan, 430060 Hubei China; 2 Institute of Urologic Disease, Renmin Hospital, Wuhan University, Wuhan, 430060 Hubei China

**Keywords:** Basement membrane, Bladder cancer, Immune, Signature, *LAMA2*

## Abstract

**Background:**

Bladder urothelial carcinoma (BLCA) is the most common genitourinary cancer and the prognosis of patients is often poor. However, studies of basement membrane-related genes (BM-related genes) in BLCA are less reported. Therefore, we established a BM-related genes signature to explore their functional and prognostic value in BLCA.

**Methods:**

In this study, a BM-related genes signature was constructed by LASSO-Cox regression analysis, and then a series of bioinformatics methods was used to assess the accuracy and validity of the signature. We constructed a nomogram for clinical application and also screened for possible therapeutic drugs. To investigate the functions and pathways affected by BM-related genes in BLCA, we performed functional enrichment analyses. In addition, we analyzed the immune cell infiltration landscape and immune checkpoint-related genes in the high and low-risk groups. Finally, we confirmed the prognostic value of BM-related genes in BLCA in vitro.

**Results:**

Combining multiple bioinformatics approaches, we identified a seven-gene signature. The accuracy and validity of this signature in predicting BLCA patients were confirmed by the test cohort. In addition, the risk score was strongly correlated with prognosis, immune checkpoint genes, drug sensitivity, and immune cell infiltration landscape. The risk score is an independent prognostic factor for BLCA patients. Further experiments revealed that all seven signature genes were differentially expressed between BLCA cell lines and normal bladder cells. Finally, overexpression of *LAMA2* inhibited the migration and invasion ability of BLCA cell lines.

**Conclusions:**

In summary, the BM-related genes signature was able to predict the prognosis of BLCA patients accurately, indicating that the BM-related genes possess great clinical value in the diagnosis and treatment of BLCA. Moreover, *LAMA2* could be a potential therapeutic target, which provides new insights into the application of the BM-related genes in BLCA patients.

**Supplementary Information:**

The online version contains supplementary material available at 10.1186/s12885-023-11340-0.

## Introduction

As one of the most common malignancies of the genitourinary system, BLCA has significant gender differences in incidence [1–3]. Meanwhile, BLCA patients who smoke and are occupationally exposed tend to have a poor prognosis [4–7]. Therefore, we urgently need new prognostic biomarkers to predict the prognosis of BLCA patients and provide new treatment ideas.

In epithelial cancers, cancer cells must pass through basement membranes (BMs) barrier to spread and metastasize. BMs are widely distributed layer of the thin, dense extracellular matrix that covers the basal surface of epithelial and endothelial cells [8]. Its core components include laminin, collagen IV, heparan sulfate proteoglycans, and nidogen, which act as structural barriers for cancer cell invasion, endocytosis, and extravasation [9]. These components are inextricably linked to biological processes of cancer cells. It has been found that abnormal expression of different structural components of BMs are associated with a variety of diseases, such as inflammation, atherosclerosis, myocardial infarction, and angiogenesis, particular tumor invasion and metastasis [10–14]. Furthermore, aberrant expression of BMs components is associated with multiple cancer types [15–19].

It is obvious that further understanding of BM-related genes is important for the progression and treatment of BLCA. However, there are few studies systematically explored the relationship between BM-related genes and BLCA. Therefore, we want to analyze the expression profile of BM-related genes in BLCA and establish a signature that can accurately predict the prognosis of BLCA patients in this study. And we further explore whether the risk score is associated with immune cell infiltration landscape, drug sensitivity, and immune checkpoint genes in BLCA patients. In conclusion, we investigated the prognostic value of BM-related genes in BLCA, aiming to provide a new perspective for the diagnosis and treatment of BLCA patients.

## Meterials and methods

### Data collection and identification of differentially expressed BM-related genes

We obtained mRNA expression and relevant clinical information for 412 BLCA samples and 19 normal bladder tissues from the Cancer Genome Atlas (TCGA, https://portal.gdc.cancer.gov/) database. 224 BM-related genes were retrieved from one previous research [11], and the specific genes are shown in Table S1. We also downloaded GSE48276 and GSE48277 including 141 BLCA samples from Gene Expression Omnibus (GEO, https://www.ncbi.nlm.nih.gov/geo/) database to validate the signature. We normalized these mRNA expression profiles and identified differentially expressed BM-related genes by the limma program package of R software with the criteria of |logFC|>1 and false discovery rate (FDR) < 0.05.

### Construction and validation of a prognostic signature based on BM-related genes

We first screened the prognostic value of BM-related genes further by univariate Cox regression analysis. Then using the R package, least absolute shrinkage and selection operator (LASSO) regression analysis was performed to construct a prognostic risk signature. The risk score was calculated by the following equation:

Risk score = ∑ (expression of gene * coef).

Where coef was the LASSO Cox regression signature coefficient for the corresponding mRNA, the specific values are shown in Table S2. Based on the median risk score, we divided BLCA patients into high-risk and low-risk groups. And we used Kaplan-Meier curves for survival analysis to assess the prognosis of the high-risk and low-risk groups. We also performed a time-dependent receiver operating characteristic (ROC) analysis to assess the prognostic predictive ability of the risk signature through the “survivalROC” R package. Finally, we selected the GEO database as a validation set to verify the accuracy of the predictive ability of the risk signature.

### Construction of a Nomogram based on risk scores and clinical variables

We investigated the relationship between risk scores based on BM-related genes signature and clinical variables. Concretely, we performed univariate and multivariate Cox regression analyses combining multiple clinical variables, with the aim of exploring whether risk scores have independent prognostic value for BLCA patients. We also used risk scores based on BM-related genes signature and clinical variables to create an outcome-related Nomogram to estimate the probability of 3- and 5-years overall survival (OS) in BLCA patients, and used the concordance index (C-index) and calibration curves to assess the predictive effect of the Nomogram.

### Functional enrichment analyses and protein-protein interactions (PPI)

We used the “ClusterProfiler” R package for Gene Ontology (GO) analysis, which includes molecular function (MF), biologic process (BP), and cellular components (CC). FDR and P < 0.05 were considered to be significantly enriched. We used STRING database (http://www.string-db.org/) to analyze the protein-protein interactions and visualized it through the Cytoscape software. We identified the most important parts of the PPI network based on MCODE scores > 10.

### Genome enrichment analysis (GSEA)

A gene set enrichment analysis (GSEA) was performed to investigate potential molecular mechanisms between the high-risk and low-risk groups. P-value < 0.05 and FDR < 25% were considered to be statistically significant.

### Immune cell infiltration analysis

There is growing evidence indicating that immune infiltration of tumor cells is involved in cancer progression and correlates with prognosis. Therefore, we used the CIBERSORT, CIBERSORT-ABS, QUANTISEQ, MCP-counter, XCELL, TIMER, and EPIC algorithms to assess the level of immune cell infiltration between high- and low-risk groups. To predict the effect of immune checkpoint therapy and to improve our understanding of the role of BM-related genes in BLCA, we explored not only the expression of several immune checkpoints, such as *LAG3*, *HAVCR2*, *CTLA4*, *PDCD1LG2*, *PDCD1*, and *TNFRSF18*, but also used the TIMER database (https://cistrome.shinyapps.io/timer/) to determine the relationship between immune cells and seven BM-related genes.

### Drug sensitivity analysis

To perform drug sensitivity analysis between high and low risk groups, we used the Genomics of Drug Sensitivity in Cancer (GDSC, http://www.cancerrxgene.org/) database to analyze the half-maximal inhibitory concentration (IC50) and predict drug sensitivity by the “pRRophetic” R package. P < 0.05 was considered statistically significant.

### Cell lines and cell culture

All cell lines were acquired from ATCC. SV-HUC-1 cell line was cultured with Ham’s F-12 K/10% fetal bovine serum media (cytiva, gibco) while BLCA cell lines (5637, T24, RT4) were cultured with RPMI 1640/10% fetal bovine serum (cytiva, gibco) media. All cells were cultured in an incubator with 5% CO2 at 37 °C.

### Transfection

The *LAMA2* plasmid and vector plasmid were purchased from Sangon Biotech (Shanghai, China). BLCA cells were seeded at the appropriate density in 6-well plates. After 12 h, transfection was performed using Lipofectamine 3000 (L3000001, Thermo Fisher Scientific, USA) according to the manufacturer’s instructions.

### Wound healing assay

BLCA cells are inoculated at the appropriate density into 6-well plates. When the cell density reaches 90–95%, a straight slit is scored on the cell surface. Wash 3 times with phosphate-buffered saline (PBS) and record the width of the gap at 0 and 24 h by using light microscope.

### Transwell invasion assay

BLCA cells (3 × 104 cells/well) were inoculated into the upper chamber and cultured in serum-free RPMI 1640 medium, and complete medium containing 10% FBS was added to the lower chamber. 24-well plates were incubated for 24 h at 37 °C, and migrating cells in the lower chamber were fixed with 4% paraformaldehyde for 30 min at room temperature and stained with 0.1% crystal violet for 20 min. Migrated cells were washed with PBS and then observed under an orthogonal microscope (Olympus, Japan).

### Western blotting assay

Samples of both tissues and cells were lysed in RIPA lysis buffer containing protease inhibitors. Proteins of different molecular weights were separated by SDS-PAGE. Transferred to a polyvinylidene fluoride (PVDF) membrane, the proteins of each sample were blocked with 5% skimmed milk for 1 h. The membranes were incubated with the primary antibodies targeting *LAMA2* (ab236762, Abcam, UK), *E-cadherin* (ab76319, Abcam, UK), *N-cadherin* (ab76011, Abcam, UK), *Vimentin* (ab8069, Abcam, UK), and *GAPDH* (ab8245, Abcam, UK). After incubation with primary antibody overnight, the membrane was washed with TBST and incubated with secondary antibody. Protein bands were visualized using an enhanced chemiluminescence reagent (WP20005, Thermo Fisher Scientific, USA). Finally, densitometric analysis was performed using ImageJ software to quantify differences in protein levels.

### Quantitative real-time polymerase chain reaction (qRT-PCR)

We extracted RNA from cells using TRIzol reagent (Thermo Fisher Scientific, USA) and PrimeScriptTM RT kit (TaKaRa, Japan) and then reverse transcribed them into cDNA. subsequently, we used TB Green PCT Master Mix (akara, Japan) for Real-time PCR for relative quantification, and *GAPDH* was selected as the experimental reference. Finally, qRT-PCR analysis was performed by the CFX96 real-time PCR system. All primers were synthesized by Sangon Biotech (Shanghai, China), and the specific sequences are shown in Table S3.

### Statistical analysis

All statistical analyses were performed with R software (version 4.0.5). The Wilcoxon test was used to compare the differences between the two groups in the high-risk and low-risk groups. P-value < 0.05 were considered statistically significant. The symbol * indicates p < 0.05, the symbol ** represents p < 0.01, and the symbol *** indicates p < 0.001.

## Results

### Establishment and validation of a signature based on BM-related genes

**Fig. 1:**
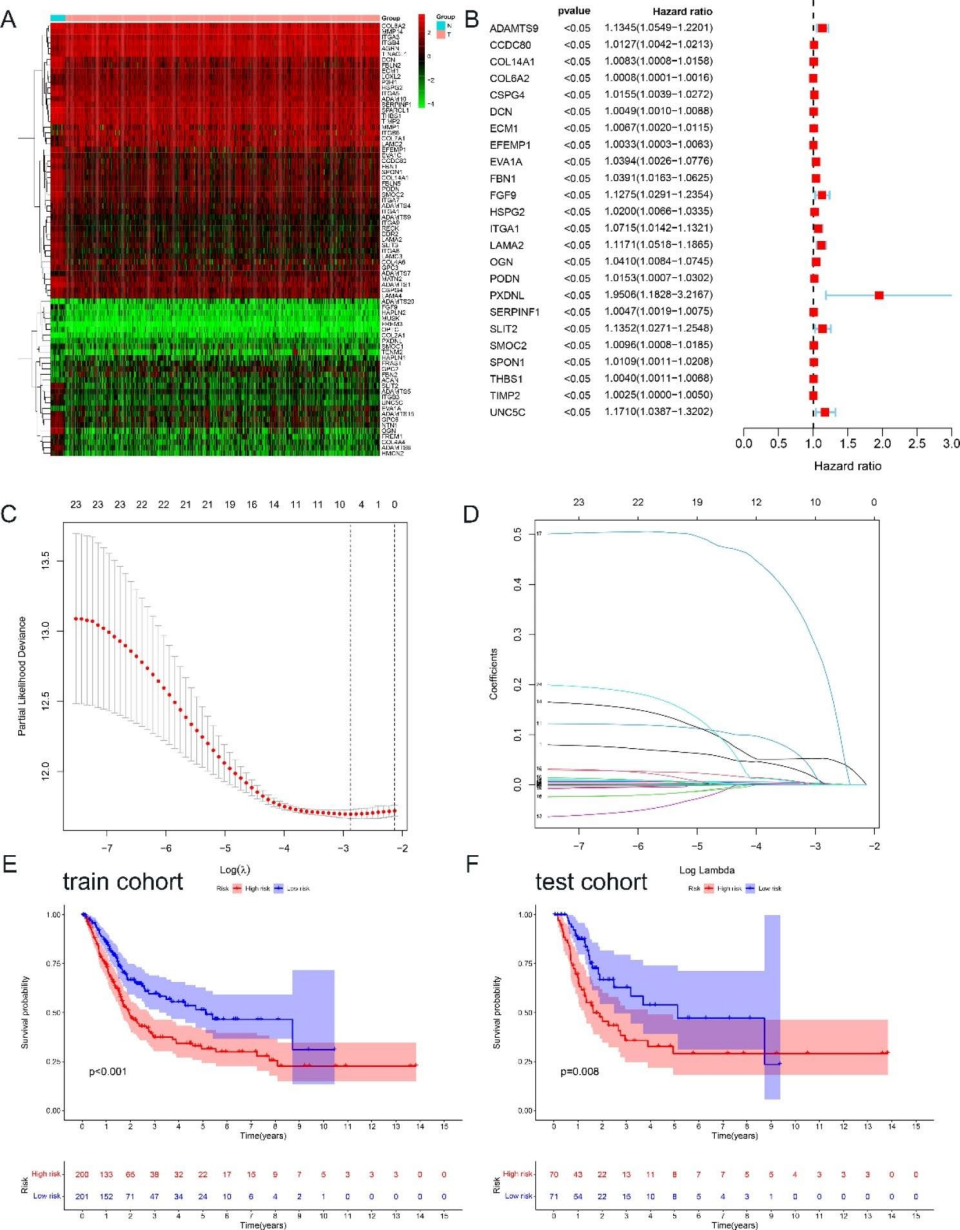
Identification and validation of BM-related genes signature. **(A)** The heat map exhibited differentially expressed BM-related genes. **(B)** Univariate Cox regression analysis showed the prognostic values. **(C**, **D)** Identification of 7 differentially expressed prognostic BM-related genes. **(E)** Kaplan-Meier curves showed the effect of the signature to predict OS in the TCGA cohort. **(F)** Verification of the signature in the GEO cohort

A total of 77 BM-related genes were identified as differentially expressed genes (DEGs) based on the TCGA-BLCA dataset, including 25 up-regulated and 52 down-regulated BM-related genes (Fig. 1A, Table S4). For these differentially expressed BM-related genes, we used univariate Cox regression analysis to assess the prognostic value of these BM-related genes. The results showed that only 24 of them had prognostic value (Fig. 1B). We then used LASSO Cox regression analysis to construct a signature that predicted the prognosis of BLCA patients. Seven genes were finally identified (*ADAMTS9, CSPG4, ECM1, FBN1, LAMA2, PXDNL* and *SERPINF1*) to establish the signature (Fig. 1C and D). The risk coefficients were calculated from the correlation coefficients of the seven BM-related genes with the following formula:

Risk score = (0.00414* *ADAMTS9* expression) + (0.00222* *CSPG4* expression) + (0.00172* *ECM1* expression) + (0.00264* *FBN1* expression) + (0.05274* *LAMA2* expression) + (0.24454* *PXDNL* expression) + (0.00004* *SERPINF1* expression).

**Fig. 2:**
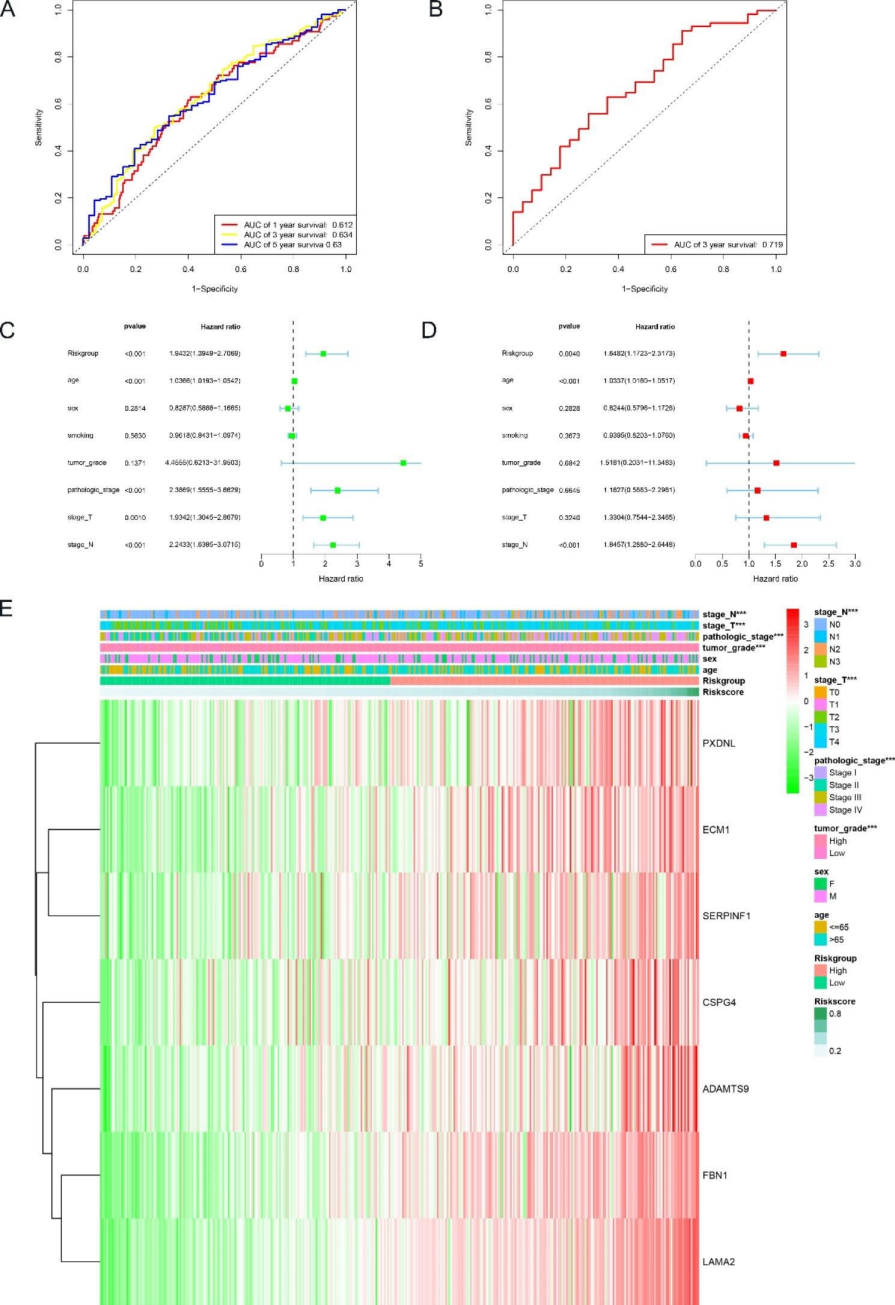
Accuracy and validity of the seven BM-related genes signature to predict the prognosis of BLCA patients. **(A)** AUC of ROC curves at 1-years, 3-years, and 5-years respectively demonstrated the predictive power of the signature in the TCGA cohort. **(B)** AUC of ROC curves at 3-years demonstrated the predictive power of the signature in the GEO cohort. **(C)** Univariate Cox regression analysis showed the correlation between clinicopathological factors and prognosis. **(D)** Multivariate Cox regression analysis showed the correlation between clinicopathological factors and prognosis. **(E)** Heatmap showed the differences of seven BM-related genes expression. The symbol * indicates p < 0.05, the symbol ** represents p < 0.01, and the symbol *** indicates p < 0.001

Based on the median risk score, BLCA patients were divided into two groups, the high-risk group and the low-risk group. As we can see, the number of deaths in the high-risk group was significantly higher than the number of deaths in the low-risk group (p < 0.001), indicating that the risk score was negatively correlated with the prognosis of BLCA patients (Fig. 1E and Figure S1A, C, E). Among the TCGA dataset, the time-dependent ROC curve showed that the AUCs based on the BM-related genes signature predicted 1-years survival, 3-years survival, and 5-years survival of 0.612, 0.634 and 0.630, respectively (Fig. 2A). We also validated the prediction of the BM-related genes signature in the GEO dataset using the same approach, and the results were consistent with the TCGA dataset (Fig. 1F and Figure S1B, D, F), with the time-dependent ROC curve showing that the AUC for predicting 3-years survival was 0.719 (Fig. 2B).

### Signature based on BM-related genes is an independent indicator of BLCA patients

Univariate and multivariate Cox analyses were performed to determine whether the signature could be an independent prognostic indicator. Univariate Cox analysis showed that risk group, age, pathologic stage, stage T and stage N were significantly associated with the prognosis of BLCA patients (p < 0.001) (Fig. 2C); multivariate Cox analysis showed that risk group, age and stage N were still significantly associated with the prognosis of BLCA patients (p < 0.05) (Fig. 2D). These results conclusively demonstrate that the signature based on BM-related genes is an independent indicator of prognosis in BLCA patients.

### Association between signature and clinical features

**Fig. 3:**
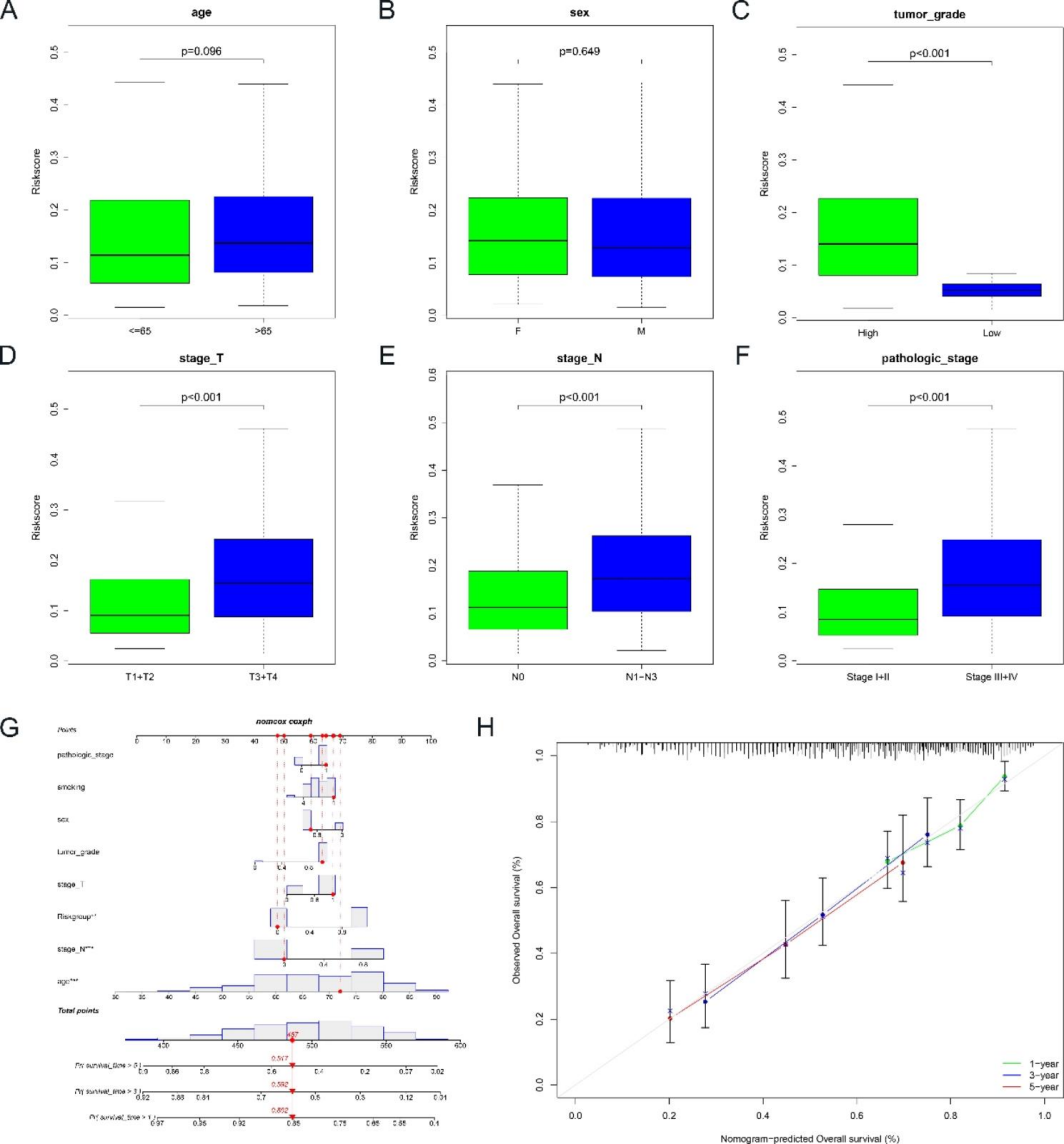
The correlation between clinicopathological factors and the signature. **(A**-**F)** Box plots showed the risk score of clinicopathological factors. **(G)** Nomogram for predicting 1-years, 3-years, and 5-years prognosis. **(H)** Calibration curve for the nomogram

The Chi-square test was used to investigate whether the prognostic signature was associated with the development and progression of BLCA. The results showed that there was a significant difference between the high-risk and low-risk groups in terms of tumor grade, stage T, stage N and pathologic stage (p < 0.001), but not in terms of age and gender (p > 0.05) (Figs. 2E and 3A-F). Subsequently, we found the prognostic significance of this signature in the subgroup by further stratification analysis. The findings showed that the BM-related genes signature performed well in predicting prognosis in the following populations: age less than or equal to 65 years (p < 0.001), age greater than 65 years (p = 0.026), male (p < 0.001), stage III + IV (p = 0.018),T3 + T4 (p = 0.009),N0 (p = 0.006),N1-N3 (p = 0.036) and high grade (p < 0.001). In contrast, the BM-related genes signature performed poorly in predicting prognosis in the following populations: female, stage I + II, T1 + T2 (Figure S2A-G).

### Construction of the Nomogram

The Nomogram combines various prognostic indicators to graphically assess the probability of survival of an individual. Therefore, a Nomogram consisting of pathologic stage, smoking, sex, tumor grade, stage T, risk group, stage N and age was constructed to predict the survival rates of BLCA patients at 1, 3 and 5 years (Fig. 3G). The calibration curve showed that the observed survival condition of the patients was consistent with the survival condition predicted by Nomogram (Fig. 3H). The C-index of the Nomogram was 0.684, which confirms the good predictive power of the Nomogram.

### Functional enrichment analyses and protein-protein interactions (PPI)

**Fig. 4:**
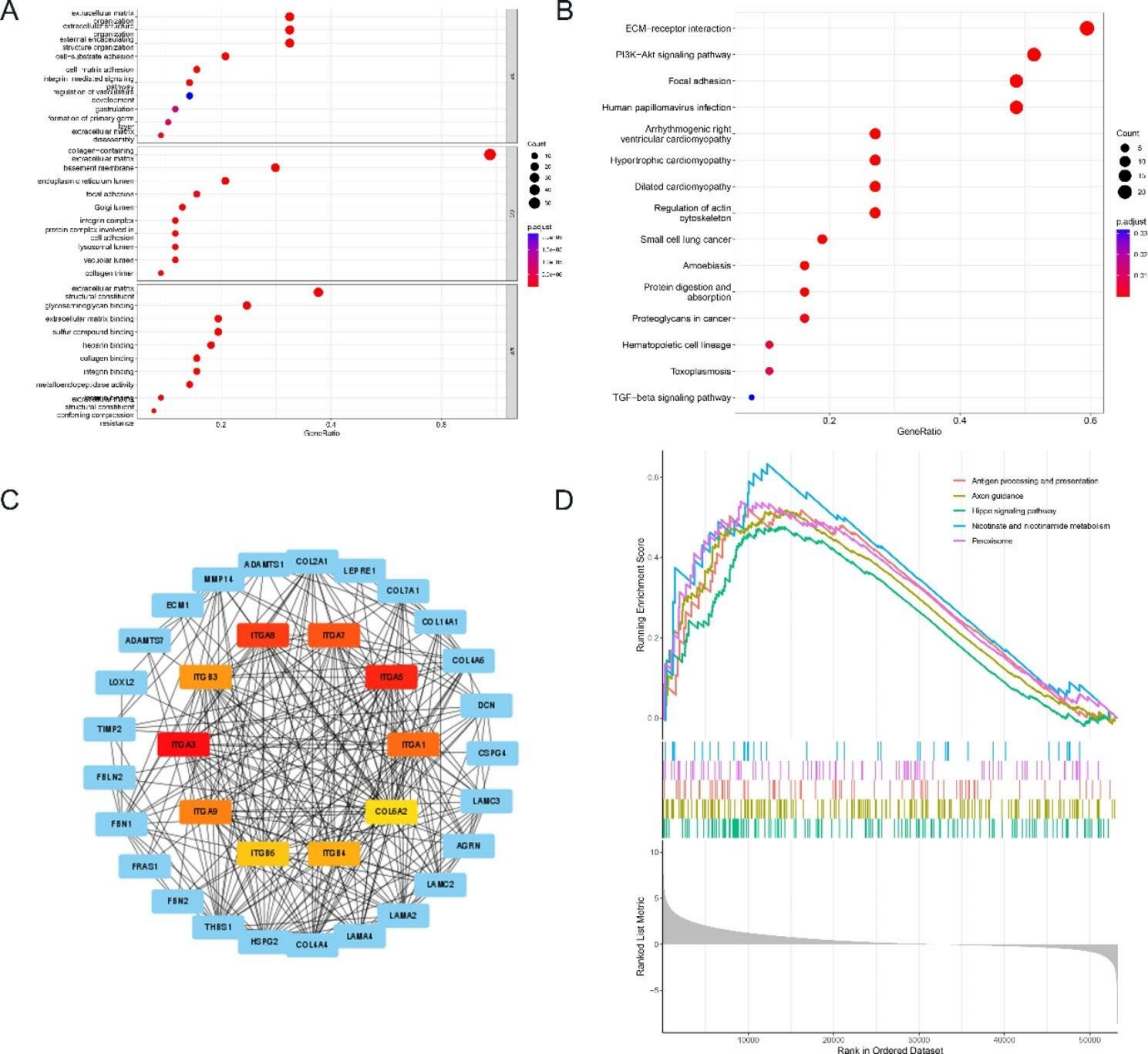
Functional enrichment and PPI analysis based on DEGs. **(A)** GO analysis. **(B)** KEGG analysis. **(C)** PPI network. **(D)** GSEA analysis

Through GO and KEGG analyses, we explored the potential functions of the differentially expressed BM-related genes. In GO analysis, the results of biological process analysis showed that 77 BM-related genes were mainly involved in extracellular matrix organization, extracellular structure organization, external encapsulating structure organization, cell-substrate adhesion, cell-matrix adhesion, integrin-mediated signaling pathway, regulation of vasculature development and extracellular matrix disassembly processes. Cellular component analysis showed that 77 BM-related genes were clearly present in collagen-containing extracellular matrix, basement membrane, focal adhesion, integrin complex, protein complex involved in cell adhesion and collagen trimer. Molecular functional analysis indicated that 77 BM-related genes were mainly located in extracellular matrix structural constituent, glycosaminoglycan binding, extracellular matrix binding, sulfur compound binding, heparin binding, collagen binding, integrin binding, metalloendopeptidase activity, laminin binding and conferring compression resistance (Fig. 4A). In the KEGG analysis, the results showed that these genes were mainly involved in ECM-receptor interaction, PI3K-Akt signaling pathway, focal adhesion, proteoglycans in cancer and TGF-beta signaling pathway (Fig. 4B). We identified 10 hub genes by constructing a PPI network based on the STRING database (Fig. 4C).

### GSEA analysis

We used GSEA analysis to further unravel the molecular mechanisms underlying the signature of BM-related genes. The results showed that antigen processing and presentation, axon guidance, Hippo signaling pathway, nicotinate and nicotinamide metabolism and peroxisome were mainly enriched in the high-risk group (Fig. 4D).

### Analysis of immune correlation based on the signature of BM-related genes

**Fig. 5:**
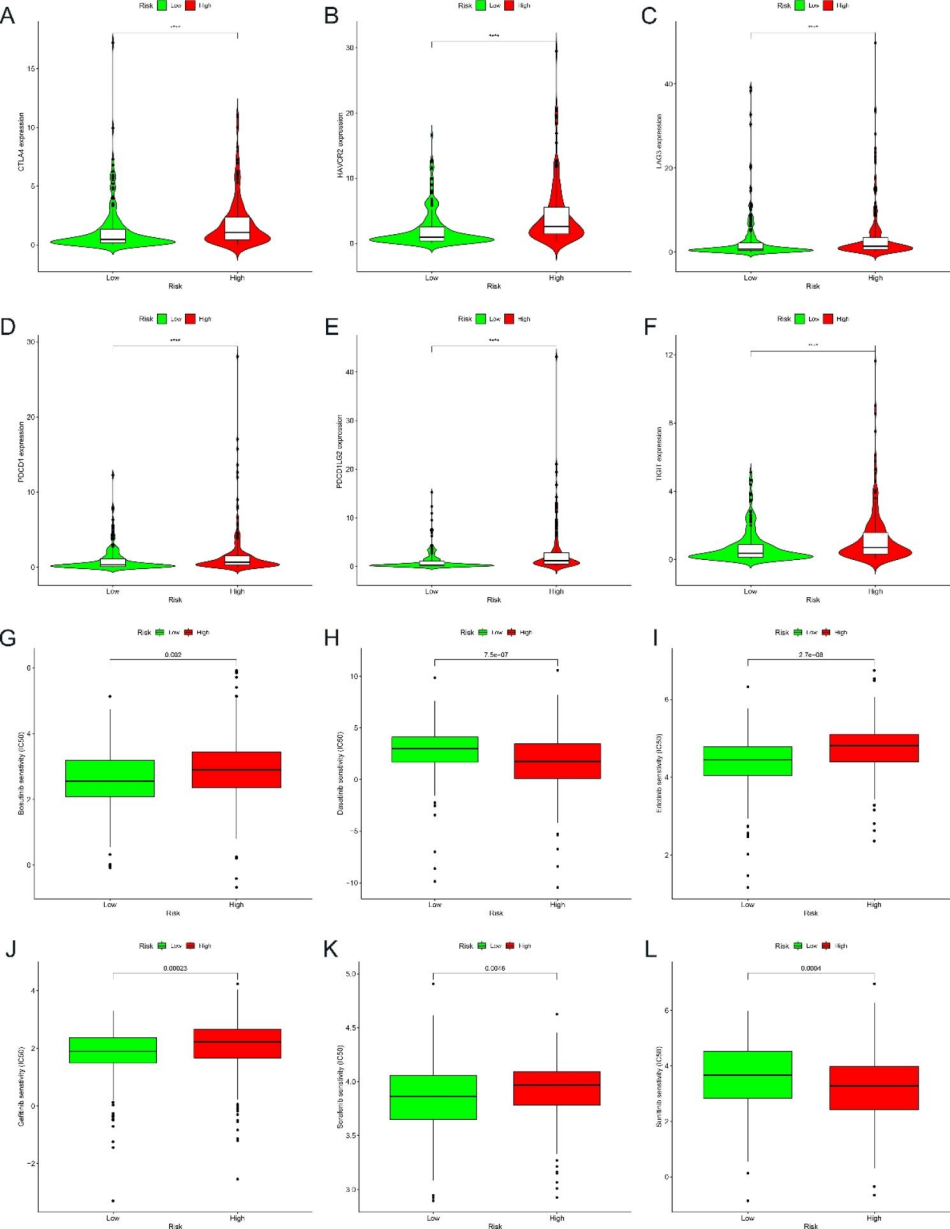
Differences in immune checkpoint genes and drug sensitivity between high and low-risk groups. (A-F) The differences in the expressions of immune checkpoint genes between high and low-risk groups. (G-L) Drug sensitivity analysis between high and low-risk groups. The symbol * indicates p < 0.05, the symbol ** represents p < 0.01, and the symbol *** indicates p < 0.001

The relationship between the signature and immune infiltration was shown in a heat map based on the results obtained from the analysis of TIMER, CIBERSORT, CIBERSORT-ABS, QUANTISEQ, MCPCOUNTER, XCELL and EPIC (Figure S1G). To provide some theoretical basis for immune checkpoint treatment strategies in bladder cancer, we also explored the expression difference of key immune checkpoints (*LAG3, HAVCR2, CTLA4, PDCD1LG2, PDCD1, TNFRSF18, TNFRSF9, TNFRSF4, TNFSF4, TNFSF18, TNFSF9* and *TIGIT*) between the high- and low-risk groups. The results showed that the expression of *LAG3, HAVCR2, CTLA4, PDCD1LG2, PDCD1, TNFRSF18, TNFRSF9, TNFRSF4, TNFSF4, TNFSF18, TNFSF9* and *TIGIT* were all elevated in the high-risk group, which was significantly different and statistically significant compared to the low-risk group (p < 0.001), indicating the presence of immunosuppressive potential in the high-risk group (Fig. 5A-F and Figure S3A-F).

### Drug sensitivity analysis

In order to further improve the treatment outcome and prolong the survival of BLCA patients, we immediately investigated the difference in sensitivity to some commonly used chemotherapeutic agents and used the IC50 values of the drugs between the high-risk and low-risk groups as an indication of drug sensitivity. Results from the GDSC database showed that patients in the high-risk group had lower IC50 values than those in the low-risk group for drugs including Dasatinib, Cisplatin, Bexarotene, Pazopanib, Parthenolide, Midostaurin and Sunitinib, suggesting that patients in the high-risk group were more sensitive to these drugs. In contrast, patients in the low-risk group had lower IC50 values than the high-risk group for drugs including Bosutinib, Gefitinib, Erlotinib, Methotrexate, Sorafenib, Salubrinal, Vinorelbine and Tipifarnib, indicating that patients in the low-risk group were more sensitive to these drugs (Fig. 5G-L and Figure S3G-O).

### TIMER analysis

**Fig. 6:**
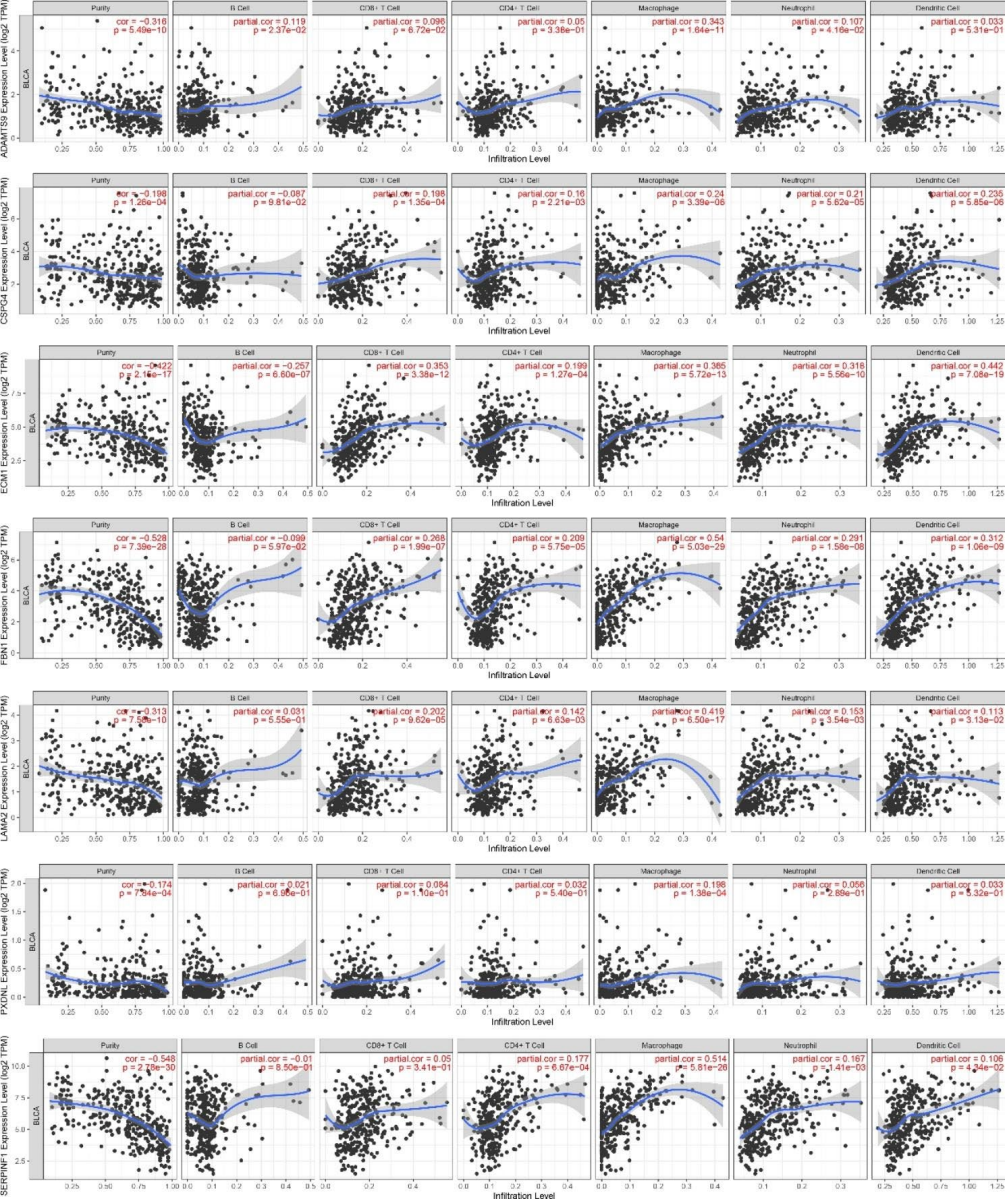
TIMER analysis for seven BM-related genes

Using the TIMER database, we investigated the relationship between immune cells and seven BM-related genes. The results showed that *ADAMTS9* was positively associated with B cells, macrophages and neutrophils. *CSPG4, ECM1, FBN1* and *LAMA2* were positively associated with a variety of immune cells such as CD8 + T cells, CD4 + T cells, macrophages, neutrophils and dendritic cells, but *ECM1* was also negatively associated with B cells. *PXDNL* was positively associated with macrophages. *SERPINF1* was positively correlated with CD4 + T cells, macrophages, neutrophils and dendritic cells (Fig. 6).

### Expression of seven prognostic BM-related genes in BLCA

**Fig. 7:**
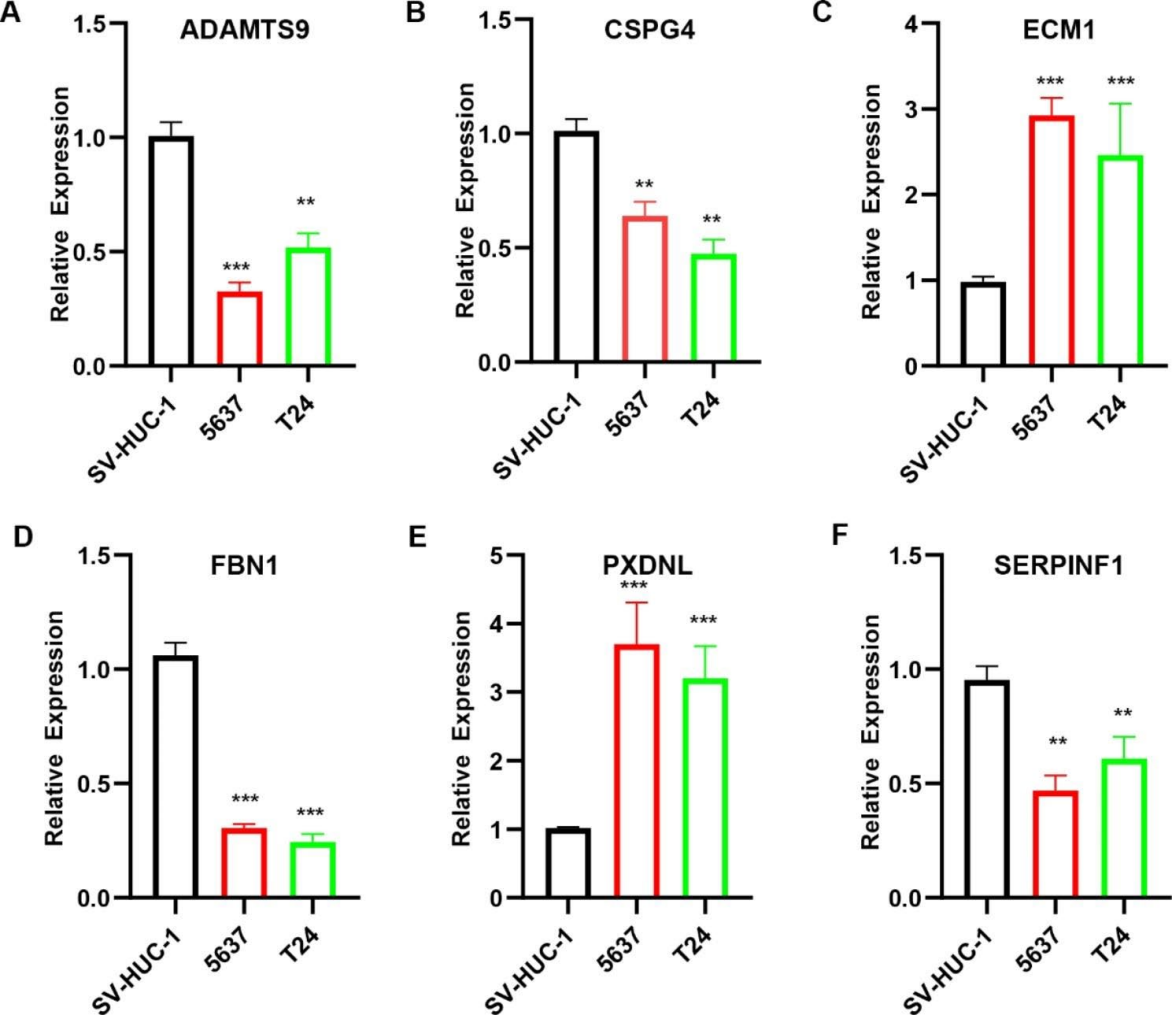
BM-related genes were differentially expressed in BLCA cell lines. The symbol * indicates p < 0.05, the symbol ** represents p < 0.01, and the symbol *** indicates p < 0.001

The results of qRT-PCR showed that seven BM-related genes were differentially expressed between BLCA cell lines and normal bladder cell lines. Among them, *ADAMTS9, CSPG4, FBN1, SERPINF1, LAMA2* were lowly expressed in BLCA cell lines and *ECM1, PXDNL* were highly expressed in BLCA cell lines (Fig. 7A-F and Figure S2H). To further verify the role of BM-related genes in BLCA, we chose *LAMA2* for further experiments.

### ***LAMA2*** is down-regulated in BLCA tissues that inhibits the migration and invasion of BLCA cell lines

**Fig. 8:**
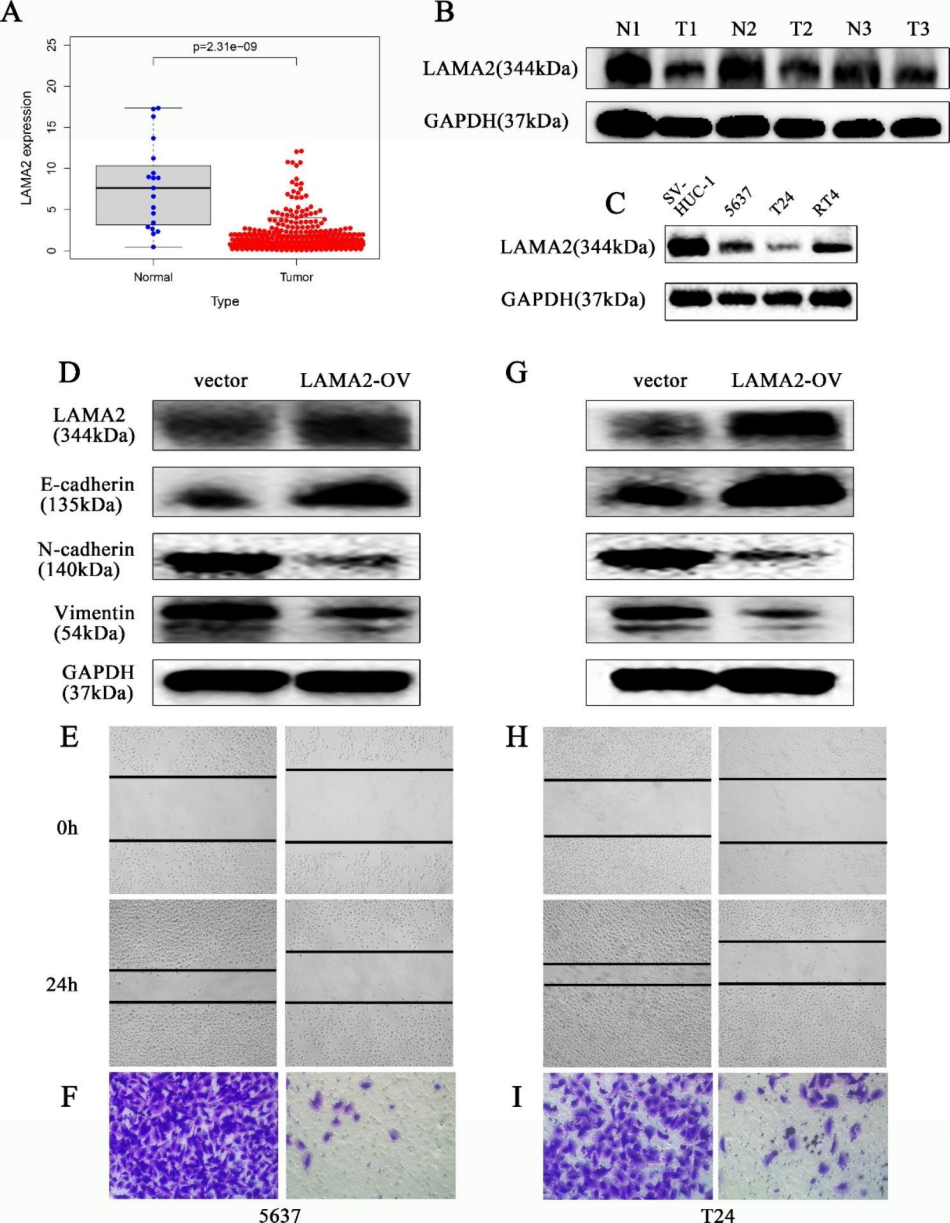
Cell experiments confirm that overexpression of *LAMA2* inhibits the migration and invasion ability of BLCA cell lines. **(A)** The difference in expression of *LAMA2* between normal and BLCA patients. **(B**, **C)** Western blotting assay showed the expressions of *LAMA2* in BLCA tissues and BLCA cell lines. **(D**-**F)** Altered levels of EMT-related markers and the ability of migration and invasion after *LAMA2* overexpression in 5637 cells. **(G**-**I)** Altered levels of EMT-related markers and the ability of migration and invasion after *LAMA2* overexpression in T24 cells

To clarify the expression of *LAMA2* in BLCA tissues, we first investigated the expression of *LAMA2* in BLCA and normal tissues. As we can see, *LAMA2* expression was significantly lower in tumor tissues than in normal tissues (Fig. 8A). We subsequently verified the expression by western blotting assay on both tumor and normal tissues and could see that *LAMA2* expression was significantly lower in tumor tissues than in normal tissues (Fig. 8B). In addition, we also analyzed the expression of *LAMA2* in SV-HUC-1 and BLCA cell lines (5637, T24, RT4), which showed that the expression of *LAMA2* in BLCA cell lines was significantly lower than that in SV-HUC-1 (Fig. 8C), and selected 5637 and T24 cell lines in the subsequent cell experiments. Overexpression of *LAMA2* in BLCA cell lines elevated the expression of *E-cadherin*, while decreasing the expression of *N-cadherin* and *Vimentin*, compared to controls (Fig. 8D, G). In further cellular assays, wound healing assay (Fig. 8E, H) and transwell invasion assay (Fig. 8F, I) showed that *LAMA2* overexpression inhibited the migration and invasion ability of BLCA cell lines. The above results indicate that *LAMA2* expression can regulate the migration and invasion ability of BLCA cell lines, which suggests that it can be a potential therapeutic gene.

## Discussion

A growing number of studies have confirmed that there are novel biomarkers such as mRNA, lncRNA, circRNA, and miRNA that can be used as biomarkers for diagnosing cancer and predicting cancer prognosis [20–23]. Highly accurate prognostic signatures based on RNA-binding proteins have been reported in many studies [24, 25]. LncRNA-based signatures for predicting the diagnosis and prognosis of cancer patients have also been continuously validated in several studies, such as immune-related lncRNA [26, 27] and autophagy-related lncRNA [28, 29]. In a study using a double-loop RNA-based signature as a non-invasive diagnostic marker for lung adenocarcinoma, a new model was established which distinguished tumor tissues very well from normal tissues [30]. In addition, another study identified cell-free urine miRNAs as promising biomarkers for the non-invasive detection of BLCA [31]. For the BM-related genes are rarely studied in BLCA, we constructed and validated a signature based on BM-related genes.

In this study, we first screened the TCGA database to identify 77 BM-related genes that were differentially expressed between BLCA and normal tissues. The biological pathways of the 77 BM-related genes were also systematically explored and a PPI network was constructed. Then by univariate and multivariate Cox regression analysis, we identified and validated a BM-related genes signature that was associated with BLCA prognosis. In addition, this signature had good predictive power based on the results in the training and test cohort. Furthermore, univariate and multivariate Cox analyses showed that the risk score based on 7 BM-related genes was an independent prognostic indicator for BLCA patients. Finally, we found that risk score was strongly correlated with the level of immune cell infiltration.

*ADAMTS9* is a novel cancer regulator that has been reported to be involved in a variety of cancers, such as gastric cancer [32], liver cancer [33], breast cancer [34], prostate cancer [35], and bladder cancer [36]. aberrant expression of *ADAMTS9* in a variety of cancers is closely associated with cancer proliferation, invasion, migration, and inhibition of apoptosis, and has been shown to mediate in various ways cancer development, such as regulating miRNAs and activating classical signaling pathways in cancer [37]. In bladder cancer, upregulation of *ADAMTS9-AS1* was found to be accompanied by activation of the *PI3K/AKT/mTOR* signaling pathway, whereas downregulation of *ADAMTS9-AS1* led to the opposite effect. It was shown that *ADAMTS9-AS1* promoted the proliferation and migration of bladder cancer cells and inhibited autophagy and apoptosis through the *PI3K/AKT/mTOR* pathway. Chondroitin sulfate proteoglycan 4 (*CSPG4*) is a cell surface proteoglycan that is expressed by various types of cancer cells and sarcomas, such as squamous cell carcinoma of the head and neck and breast cancer. *CSPG4* plays an important role in the growth and survival of tumor cells, and overexpression of *CSPG4* has been associated with recurrent metastasis of cancer [38–40]. Extracellular matrix protein 1 (*ECM1*) is a secreted glycoprotein that is predominantly expressed in the perivascular area but is also significantly elevated in many malignant epithelial tumors that produce metastasis [41]. One study found that *ECM1* was expressed in the human breast cancer cell lines MDA-435 and LCC15, both of which are highly tumorigenic, and the results suggest that *ECM1* has angiogenic properties and may promote tumor development [42]. There is also a study demonstrating that *ECM1* plays an important role in cancer metastasis by stabilizing *β-catenin* [43]. The role of Fibrillin-1 (*FBN1*) in cancer is unclear, with one study showing that *MiR-133b* inhibited the proliferation, migration, and invasive capacity of GC cells by increasing *FBN1* expression [44], while another article found that *FBN1* promotes cisplatin resistance in ovarian cancer by maintaining energy stress and inducing angiogenesis in vitro and in vivo [45]. *LAMA2* encodes the α2 chain, which constitutes one of the subunits of laminin 2. Downregulation of *LAMA2* has been demonstrated in a variety of cancer types, including lung adenocarcinoma, invasive PiNETs, colon cancer, and bladder cancer, suggesting that *LAMA2* is a suppressor gene. It was found that the knockdown of *LAMA2* promoted cancer cell migration, invasion, epithelial-mesenchymal transition (EMT), and activation of the PI3K/AKT pathway [46–49]. The peroxidase-like enzyme (*PXDNL*) is a member of the peroxidase gene family. This gene encodes a peroxidase-like protein. One study found a general decrease in survival in breast cancer patients with high *PXDNL* expression, and *PXDNL* could be used as a potential and independent prognostic biomarker for breast cancer [50]. However, studies on *PXDNL* are scarce and its underlying mechanisms are unclear. *SERPINF1*, also known as pigment epithelium-derived factor (*PEDF*), is a multifunctional secreted protein. *SERPINF1* inhibits tumor angiogenesis and metastasis, induces apoptosis and differentiation of tumor cells, and has antitumor effects in a variety of cancers including cervical and pancreatic cancers. Decreased levels of *SERPINF1* are associated with angiogenesis, fibrosis, inflammation, autophagy, metastasis, and prognostic deterioration in tumors, and *SERPINF1* plays a multifunctional role and has therapeutic potential in a variety of cancers [51–56].

The analysis of the differential expression of immune checkpoints between the high-risk and low-risk groups showed that the expression of *LAG3, HAVCR2, CTLA4, PDCD1LG2, PDCD1, TNFRSF18, TNFRSF9, TNFRSF4, TNFSF4, TNFSF18, TNFSF9*, and *TIGIT* was higher in the high-risk group of BLCA patients than in the low-risk group. This suggests that the immune microenvironment may be suppressed and it may contribute to the poor prognosis of BLCA patients in the high-risk group. Therefore, BLCA patients could benefit from immune checkpoint inhibitor immunotherapy. Through drug sensitivity analysis, we found that BLCA patients in the high-risk group may benefit from treatment with Dasatinib, Cisplatin, Bexarotene, Pazopanib, Parthenolide, Midostaurin and Sunitinib, and BLCA patients in the low-risk group may benefit from treatment with Bosutinib, Gefitinib, Erlotinib, Methotrexate, Sorafenib, Salubrinal, Vinorelbine and Tipifarnib. What’s more, we quantified the expression of seven BM-related genes in BLCA cell lines by qRT-PCR. Further experiments showed that overexpression of *LAMA2* inhibited the migration and invasive ability of BLCA cell lines, suggesting that *LAMA2* may be involved in the progression of BLCA. Therefore, it may be possible to delay the progression of BLCA by modulating *LAMA2* expression, offering more therapeutic possibilities for patients with BLCA.

## Conclusions

In this study, we identified seven BM-related genes to predict the prognosis of BLCA patients. And we further quantified the expression of seven BM-related genes in BLCA cell lines. In addition, vitro experiments showed that overexpression of *LAMA2* can inhibit the migration and invasion of BLCA cell lines. In summary, we have confirmed the close association between BM-related genes and BLCA patients through a series of bioinformatics approaches and cellular experiments, providing new insights into the diagnosis and treatment of BLCA patients.

### Electronic supplementary material

Below is the link to the electronic supplementary material.

Supplementary Material 1

Supplementary Material 2

Supplementary Material 3

## Data Availability

All analyzed data are included in the manuscript. The datasets used and analyzed during the current study are available from the corresponding author upon reasonable request.
