# Supplementary material for: Identification of a basement membrane-related genes signature with immune correlation in bladder urothelial carcinoma and verification in vitro
Source: BMC Cancer. 2023 Oct 23;23:1021. doi: 10.1186/s12885-023-11340-0 (PMC10591420; doi:10.1186/s12885-023-11340-0)
Supplement: Supplementary file 1 — Supplementary Material 1 [file 12885_2023_11340_MOESM1_ESM.docx]

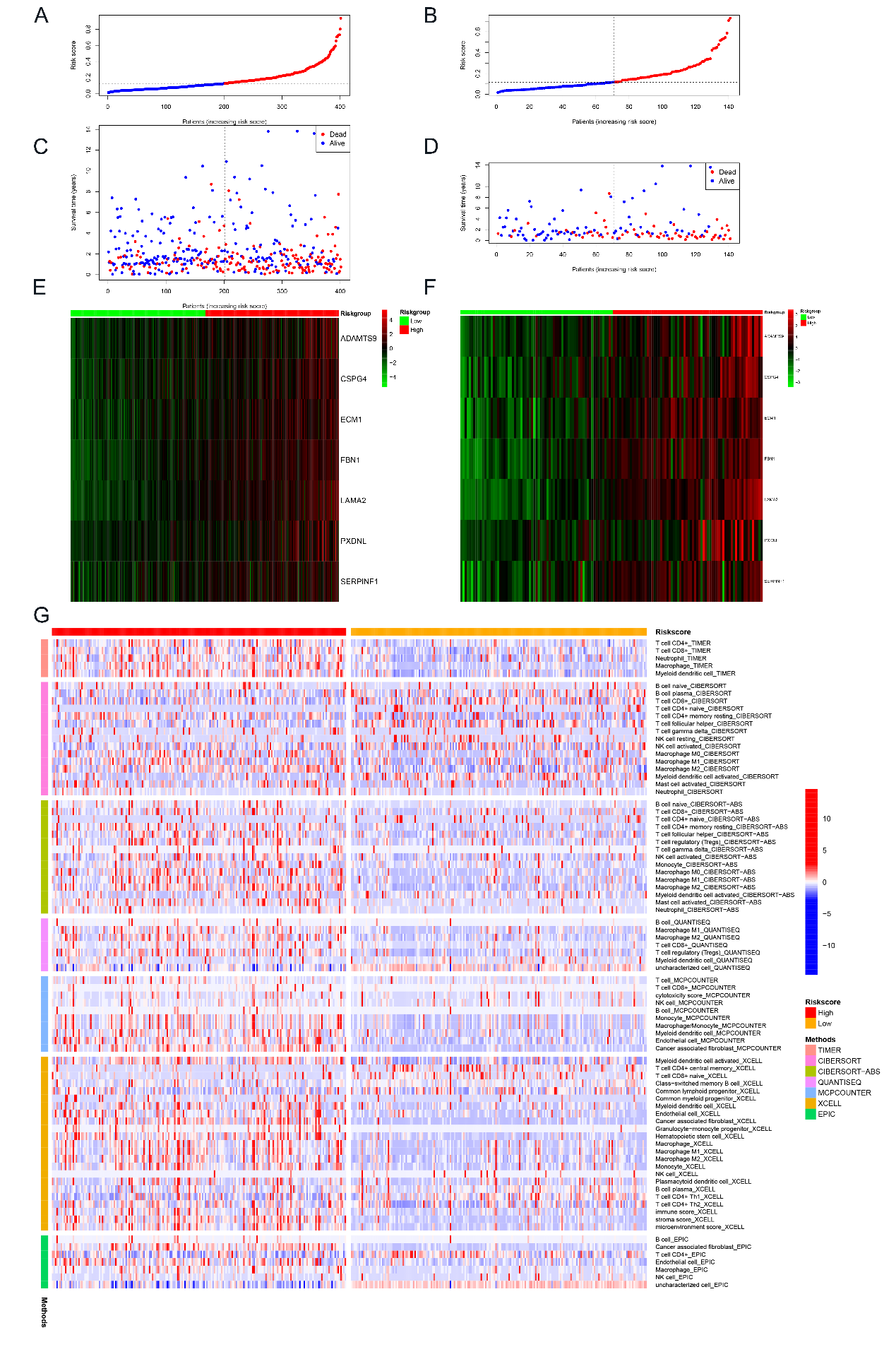


**Figure S1:** Effect of risk score on survival status, gene expression (A-F), and immune cells infiltration (G) in BLCA patients.


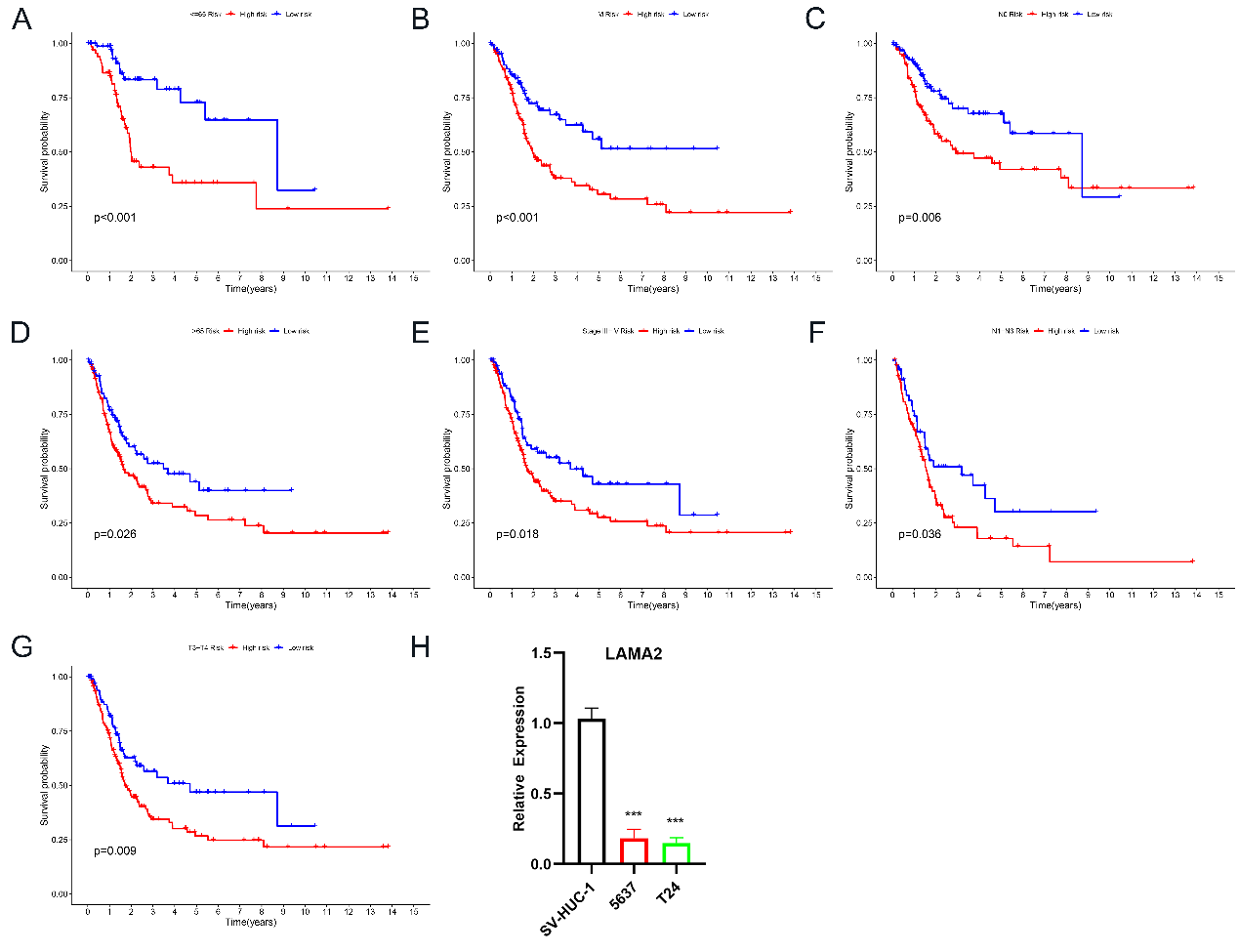


**Figure S2:** Survival analysis of clinical subgroups and differential expression of *LAMA2*. Kaplan-Meier curves showed the OS differences of clinical characteristics between different risk groups (A-G). *LAMA2* is differentially expressed between BLCA cell lines and normal bladder cells (H).
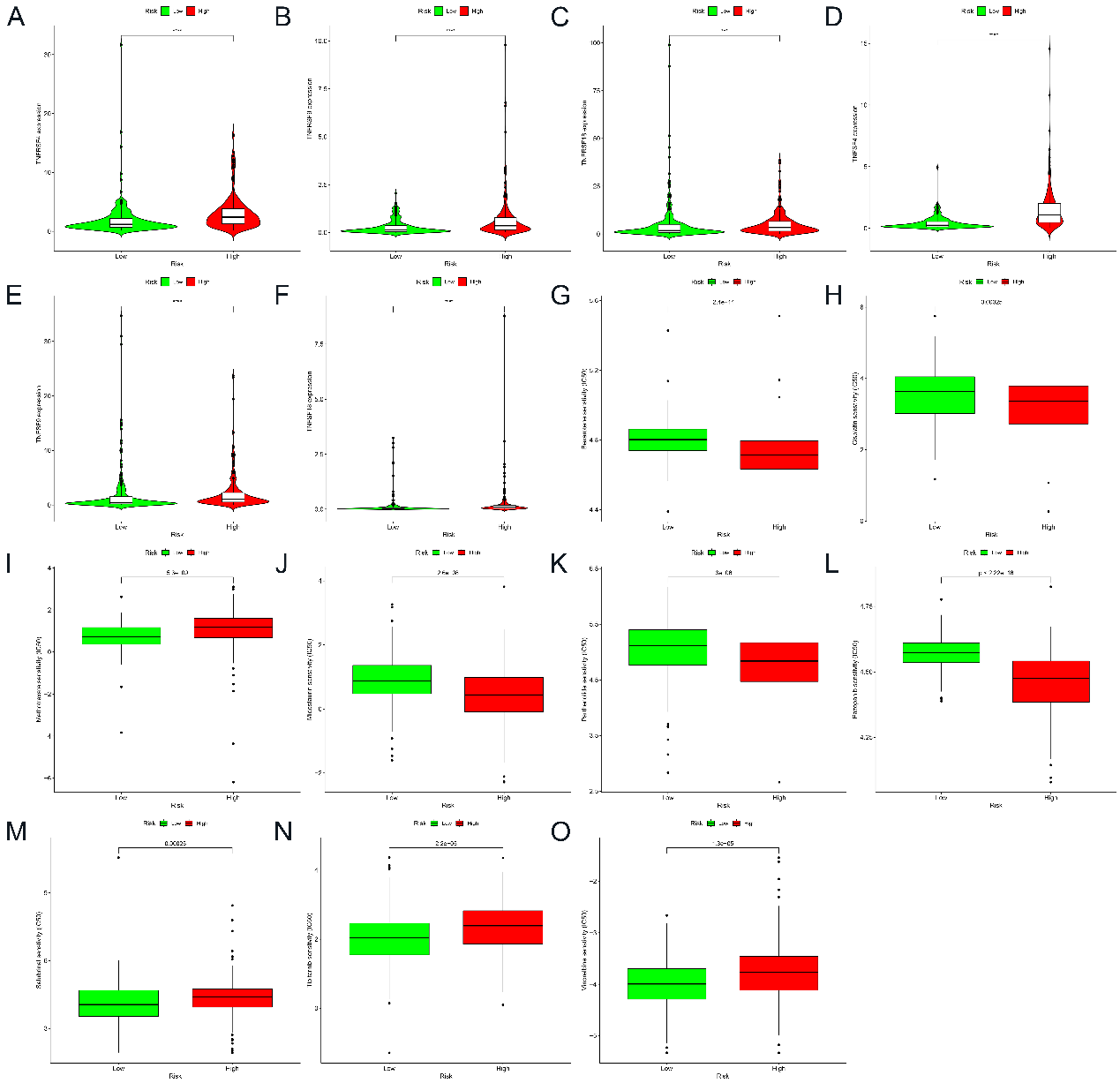


**Figure S3:** Differences in immune checkpoint genes and drug sensitivity between high and low-risk groups. (A-F) The differences in the expressions of immune checkpoint genes between high and low-risk groups. (G-O) Drug sensitivity analysis between high and low-risk groups. The symbol * indicates p<0.05, the symbol ** represents p<0.01, and the symbol *** indicates p<0.001.
