# Supplementary material for: Identification of a basement membrane-related genes signature with immune correlation in bladder urothelial carcinoma and verification in vitro
Source: BMC Cancer. 2023 Oct 23;23:1021. doi: 10.1186/s12885-023-11340-0 (PMC10591420; doi:10.1186/s12885-023-11340-0)
Supplement: Supplementary file 3 — Supplementary Material 3 [file 12885_2023_11340_MOESM3_ESM.pdf]

Figure 8B

LAMA2

GAPDH

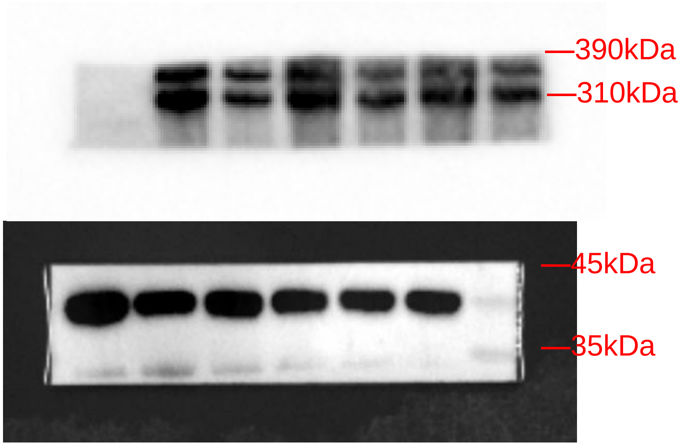

Figure 8C

LAMA2

GAPDH

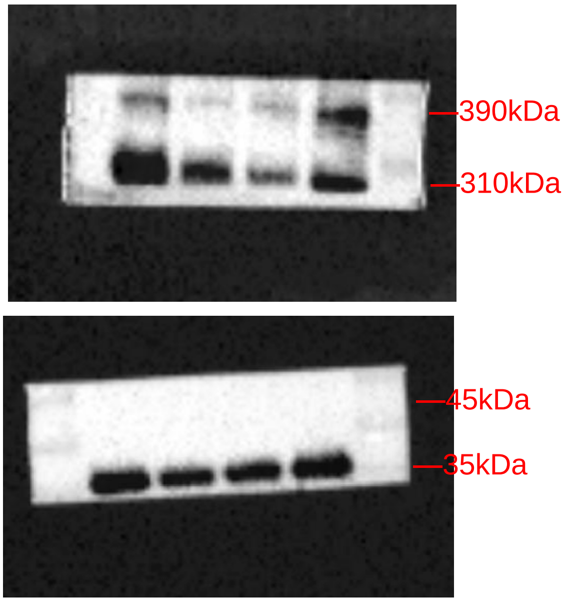

Figure 8D

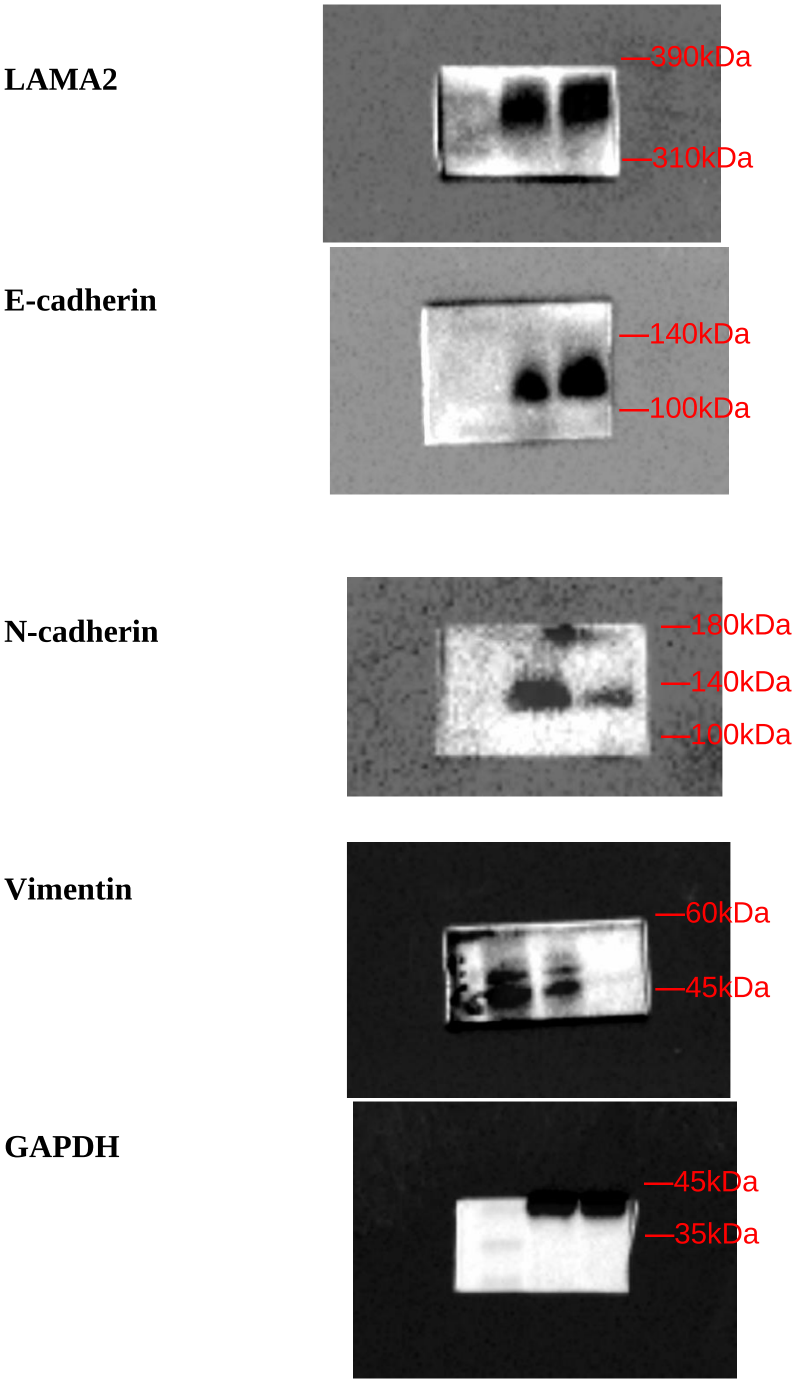
LAMA2
—390kDa
—310kDa
E-cadherin
—140kDa
—100kDa
N-cadherin
—180kDa
—140kDa
—100kDa
Vimentin
—60kDa
—45kDa
GAPDH
—45kDa
—35kDa

Figure 8G

LAMA2

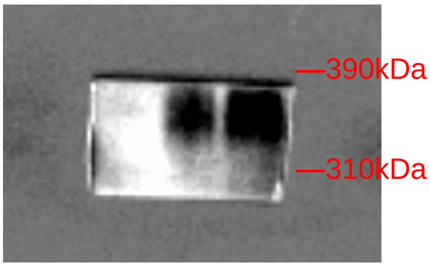

E-cadherin

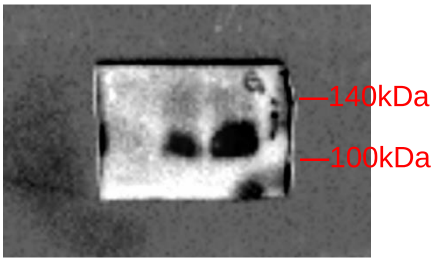

N-cadherin

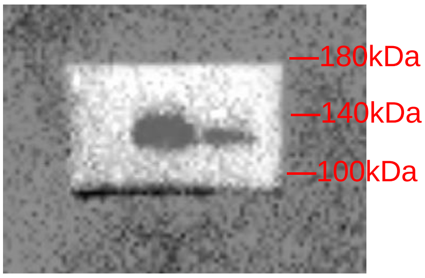

Vimentin

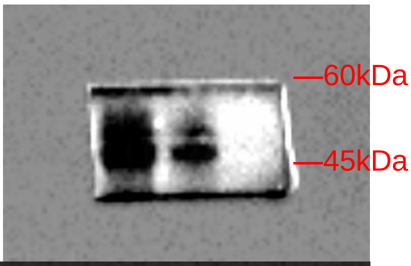

GAPDH

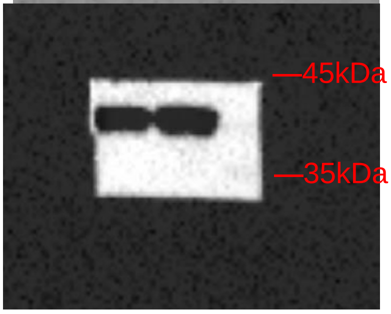

Figure 8E and H

0h-vector-5637

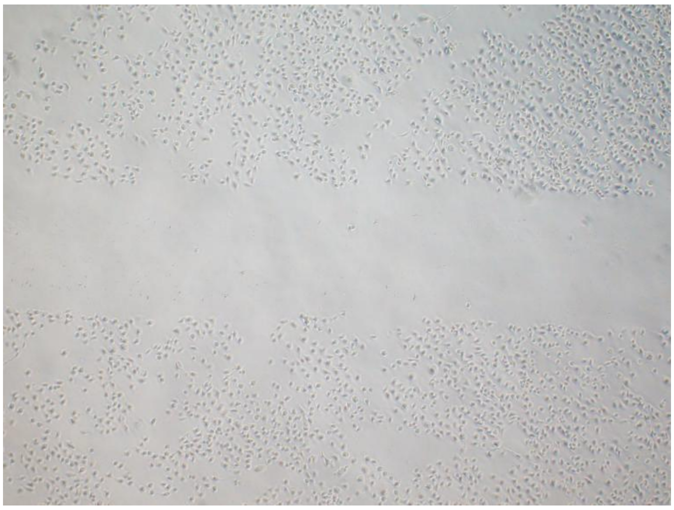

0h-vector-T24

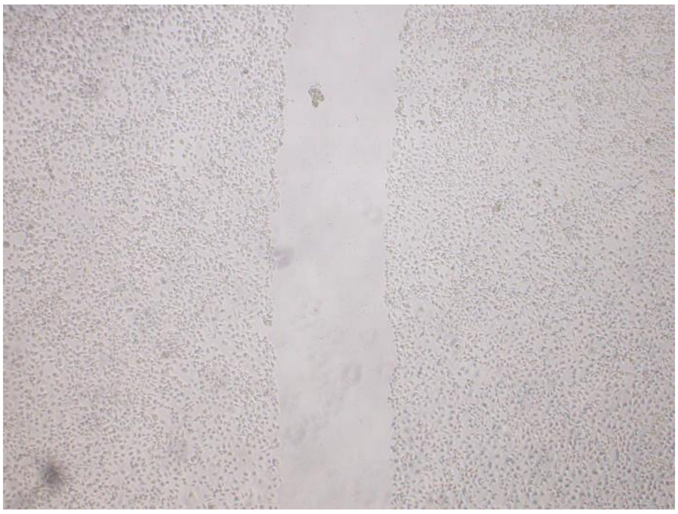

0h-OV-5637

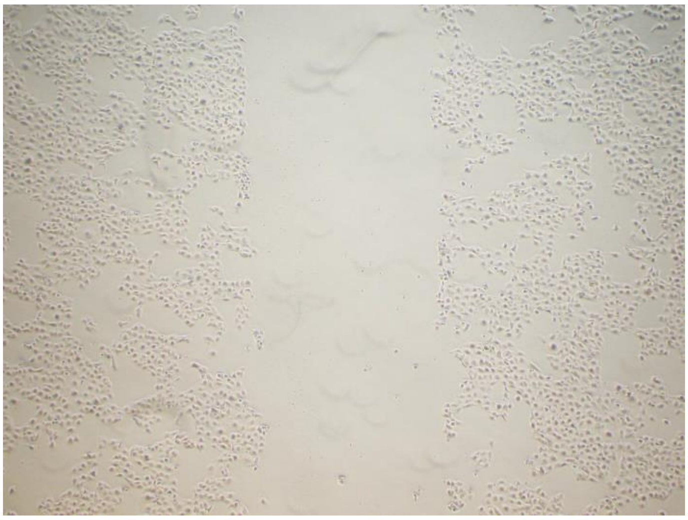

0h-OV-T24

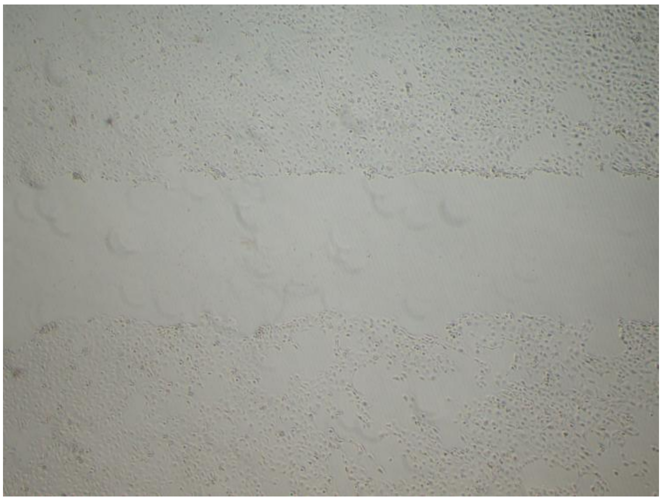

24h-vector-5637

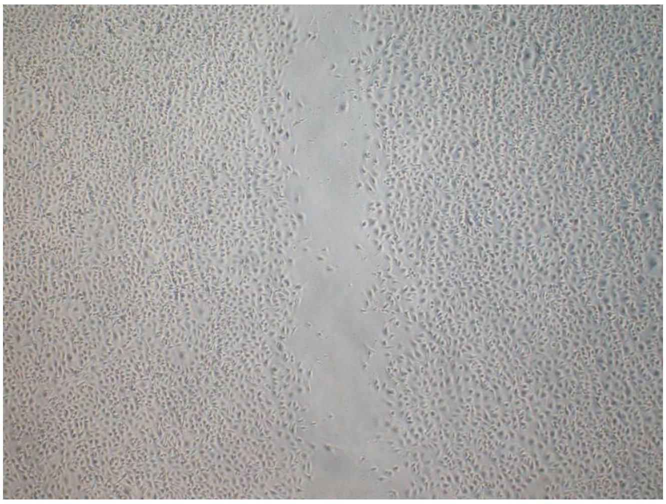

24h-vector-T24

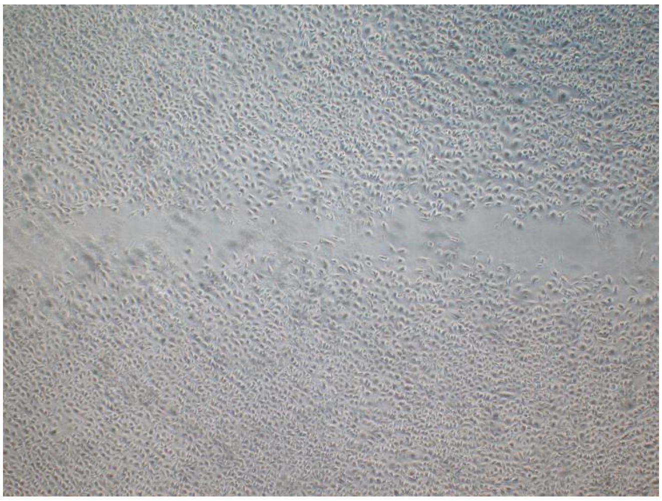

24h-OV-5637

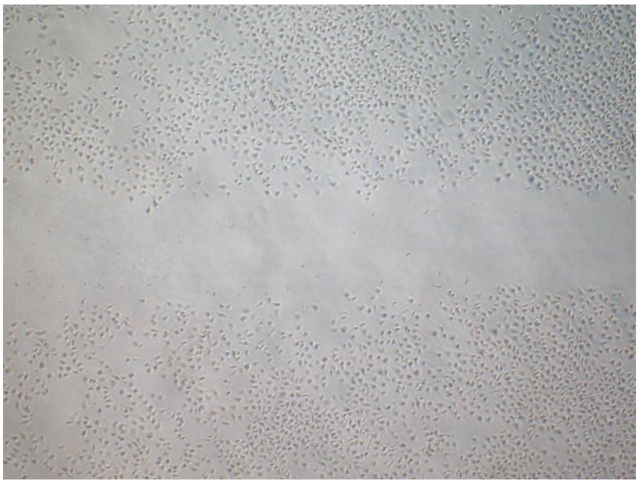

24h-OV-T24

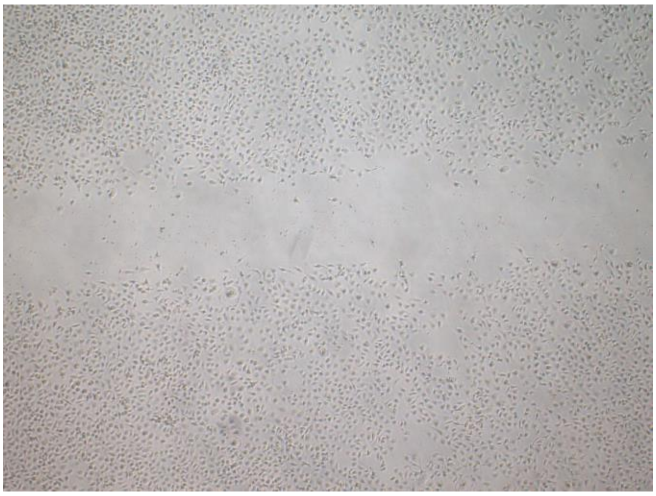

Figure 8 F

Vector

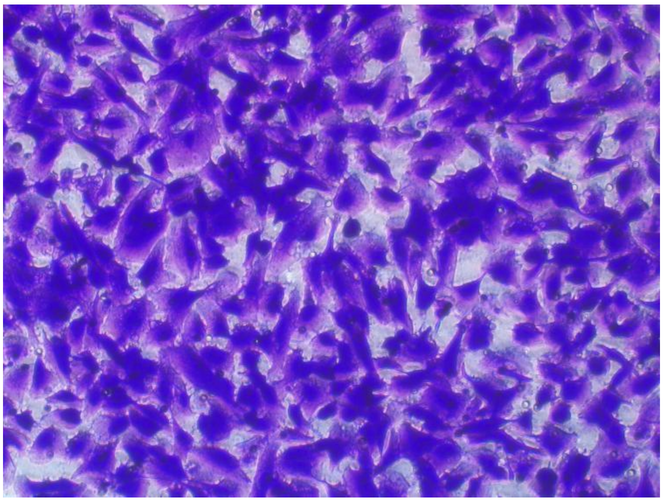

OV

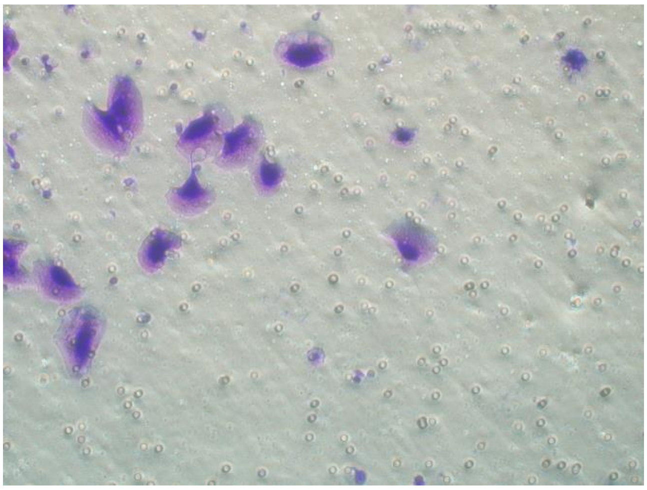

Figure 8 I

vector

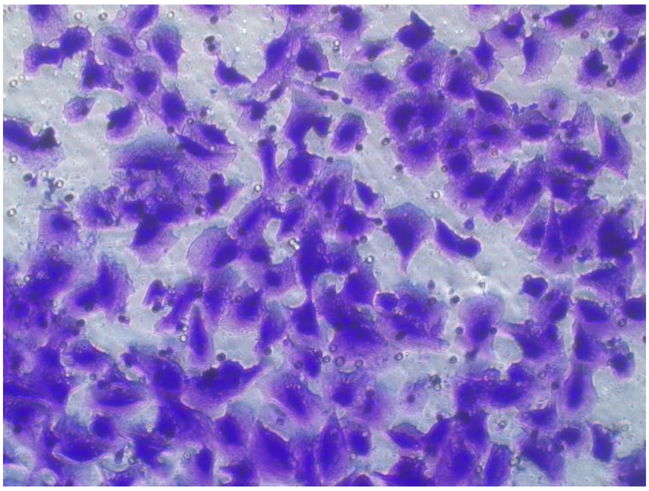

OV

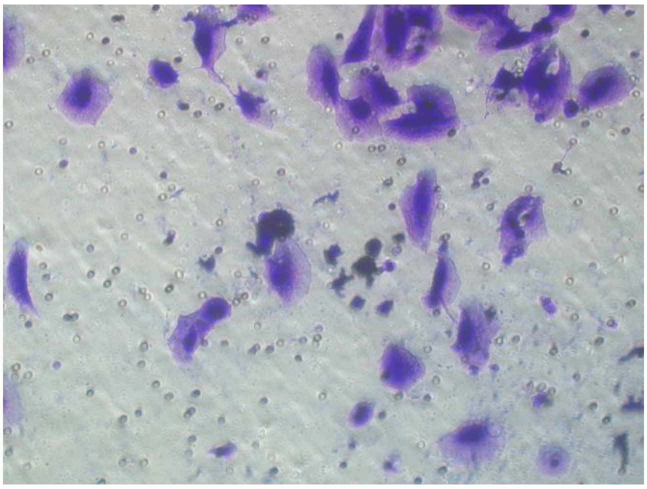

```
####鍏虫敞寰俊鍏紼紬鍙风敓淇＄姘浜哄洟闃?
###闃囧埌浠ｇ爜鎶ラ敊绛変笉鍥伝殑閹嶇   鍙  互娣诲姞寰   缂俊 scikuangren
杩涜 绛旂枒
###浣滆€呴偖绠憋細 sxkrteam@shengxinkuangren.com


install.packages("caret")



library(caret)
setwd("C:\\Users\\qqm\\Desktop\\chromatin\\24_step24")
data<-read.table("Riskscore.txt",header=T,sep="\t",check.names = F)
set.seed(666)
td<-createDataPartition(y=data$status,p=0.7,list=F)
traind<-data[td,]
rownames(traind)=traind$id
mycoef=read.table("gene_coef.txt",header = T,sep="\t")
mycoef$coef
expcoef=function(x){crossprod(as.numeric(x),mycoef$coef)}
Riskscore=apply(traind[,4:ncol(traind)],1,expcoef)
Riskgroup=as.vector(ifelse(Riskscore>median(Riskscore),"High","Low"))
newdf2=cbind(traind,Riskscore=as.vector(Riskscore),Riskgroup)
write.table(newdf2,"Riskscoretest.txt",sep="\t",quote=F,row.names=F)
```

####鍏虫敞寰俊鍏紼紬鍙风敓淇＄姘浜哄洟闃?
###闃囧埌浠ｇ爜鎶ラ敊绛変笉鍥伝殑閹嶇   鍙 互娣诲姞寰   缂俊 scikuangren
杩涜 绛旂枒
###浣滆€呴偖绠憋細 sxkrteam@shengxinkuangren.com

```
####鍏虫敞寰俊鍏綔紬鍙风敓淇＄鎷涙哄渶闃?
###闃囧埉浠ｇ爜鎶ラ敊缂变笉鎳傜殑闂蝬   鍙  互娣诲姞寰   缁俊 scikuangren
杩涜  绛旂枒
###浣滆€呴偖绠憋細 sxkrteam@shengxinkuangren.com
#library r packages
library(org.Hs.eg.db)
library(clusterProfiler)
library(enrichplot)
library(ggplot2)
setwd("C:\\Users\\qqm\\Desktop\\chromatin\\18_step18")

data3=read.table("allgene.txt",sep="\t",check.names=F,header=T)
gene_name=as.vector(data3[,1])
geneID <- mget(gene_name, org.Hs.egSYMBOL2EG, ifnotfound=NA)
geneID  <-  as.character(geneID)
data=cbind(data3,entrezID=geneID)
write.table(data,"name_id.txt",sep="\t",quote = F,row.names = F)
mydata=data[,c("entrezID","logFC")]
mydata$logFC=sort(mydata$logFC,decreasing = T)
mygenelist=as.numeric(as.character(mydata$logFC))
names(mygenelist)=as.character(mydata$entrezID)
R.utils::setOption("clusterProfiler.download.method",'auto')
mygse <- gseKEGG(geneList     = mygenelist,
                 organism     = 'hsa',keyType = "kegg",
                 pvalueCutoff = 0.05,
                 pAdjustMethod = "none" )
gse_result <- as.data.frame(mygse)
write.table(gse_result,"gse_result.txt",quote = F,sep = "\t")

gseaplot2(mygse,                    geneSetID                    =
rownames(mygse@result)[(order(mygse@result$enrichmentScore))][1:4])
```

####鍏虫敞寰俊鍏紬鍙风敓淇＄嫔浜哄洟闃?
###閬囧埌浠ｇ爜鎶ラ敊绛変笉鐭ヨ繚殑闂蝬　鍙　互娣诲姞寰　俊 scikuangren 杩涜　绛旀枒
###浣滆€呴偖绠憋細 sxkrteam@shengxinkuangren.com

```
setwd("C:\\Users\\qqm\\Desktop\\chromatin\\17_step17")
library(limma)
tcga<-read.table("mRNA.txt",header = T,sep = "\t",check.names = F)
tcga=as.matrix(tcga)
rownames(tcga)=tcga[,1]
GeneExp=tcga[,2:ncol(tcga)]
TCGA=matrix(as.numeric(as.matrix(GeneExp)),nrow=nrow(GeneExp),dimnames
=list(rownames(GeneExp),colnames(GeneExp)))
TCGA=avereps(TCGA)
TCGA=TCGA[rowMeans(TCGA)>0,]
st<- which(substr(colnames(TCGA),14,15) == '11')
tumor=TCGA[,-st]
tumor=as.data.frame(tumor)
nc=substr(colnames(tumor),1,12)
colnames(tumor)=nc
tumor=cbind(id=row.names(tumor),tumor)
#head(tumor)
tumor=t(tumor)

write.table(tumor,"tumor.txt",sep = "\t",quote = F,col.names = F)
mRNA=read.table("tumor.txt",header = T,sep = "\t",check.names =F)
risk=read.table("Riskscore.txt",header = T,sep = "\t",check.names = F)
risk_mRNA=merge(risk,mRNA,by="id")
risk_mRNA=risk_mRNA[order(risk_mRNA$Riskscore,decreasing = T),]
table(risk_mRNA$Riskscore)
risk_mRNA=t(risk_mRNA)
write.table(risk_mRNA,"risk_mRNA.txt",sep = "\t",quote = F,col.names =
F)

####
m6Agene=read.table("risk_mRNA.txt",header = T,sep = "\t",check.names =
F)
m6Agene=m6Agene[-1,]
m6Agene=as.matrix(m6Agene)
rownames(m6Agene)=m6Agene[,1]
geneE=m6Agene[,2:ncol(m6Agene)]
newgene=matrix(as.numeric(as.matrix(geneE)),nrow=nrow(geneE),
               dimnames=list(rownames(geneE),colnames(geneE)))
newdf=data.frame()
```

```
group1=255
group2=254

for(a in

row.names(newgene)){ mydata=rbind(geneexp=newgene[a,],mygroup=c(rep(1,group1)

,rep(2,group2)
))
  mydata=as.matrix(t(mydata))
  wilcoxTest<-wilcox.test(geneexp ~ mygroup, data=mydata)
  group1mea=mean(newgene[a,1:group1])
  group2mea=mean(newgene[a,(group1+1):ncol(newgene)])
  logFC=log2(group2mea)-log2(group1mea)
  p=wilcoxTest$p.value
  group1medi=median(newgene[a,1:group1])
  group2medi=median(newgene[a,(group1+1):ncol(newgene)])
  diffmedi=group2medi-group1medi
  newdf=rbind(newdf,
              cbind(gene=a,
                    group1mea=group1mea,
                    group2mea=group2mea,
                    logFC=logFC,
                    p=p))



}

p=newdf[,"p"]
FDR=p.adjust(as.numeric(as.vector(p)),method="fdr")
newdf=cbind(newdf,FDR=FDR)
newdf=na.omit(newdf)
newdf2=which(newdf$group1mea==0 | newdf$group2mea==0)
newdf3=newdf[-newdf2,]
write.table(newdf3,"allgene.txt",sep="\t",row.names=F,quote=F)

####鍏虫敞寰俊鍏綔鍙风敓淇＄嫓浜哄湪閫?
###閬囧埌浠ｇ爜鎶ラ敊绛夊笉鎳傜殑闂  鍙  互娣诲姞寰  俊 scikuangren
杩涜 绛旂枒
###浣滆€呴偖绠憋細 sxkrteam@shengxinkuangren.com
```

```
####鍏虫敞寰俊鍏粛紬鍙风敓淇＄嫔浜哄洟闃?
###閬囧埌浠ｇ爜鎶ラ敊绛变笉鐞傜殑闂鐩   鍙  互娣诲姞寰   绣俊 scikuangren
杩涜  绛旂枒
###浣滆€呴偖绠憋細 sxkrteam@shengxinkuangren.com
library(ggpubr)
library(limma)


setwd("C:\\Users\\qqm\\Desktop\\chromatin\\20_step20")
immcheck=read.table("Immune_checkpoint_genes.txt",header  =  F,sep  =
"\t",check.names = F)
tcga=read.table("mRNA.txt",header = T,sep = "\t",check.names =F)
tcga=as.matrix(tcga)
rownames(tcga)=tcga[,1]
GeneExp=tcga[,2:ncol(tcga)]
TCGA=matrix(as.numeric(as.matrix(GeneExp)),nrow=nrow(GeneExp),dimnames
=list(rownames(GeneExp),colnames(GeneExp)))
TCGA=TCGA[rowMeans(TCGA)>0,]
st<- which(substr(colnames(TCGA),14,15) == '11')
tumor=TCGA[,-st]
tumor=avereps(tumor)
tumor=as.data.frame(tumor)
nc=substr(colnames(tumor),1,12)
colnames(tumor)=nc
tumor=tumor[as.vector(immcheck[,1]),]
tumor=na.omit(tumor)
tumor=t(tumor)
tumor=cbind(id=rownames(tumor),tumor)
risk=read.table("Riskscore.txt",header = T,sep = "\t",check.names = F)
risk_mRNA=merge(risk,tumor,by="id")
for(a in
  colnames(risk_mRNA[,3:ncol(risk_mRNA)])){ risk_mRNA$Risk=fact
  or(risk_mRNA$Risk, levels=c("Low", "High"))
  mygroup=levels(factor(risk_mRNA[,"Risk"]))
  mycom=combn(mygroup, 2)
  mycomlist=list()
  for(b in 1:ncol(mycom)){mycomlist[[b]]<-mycom[,b]}
  df=data.frame(Risk=risk_mRNA$Risk,a=as.numeric(risk_mRNA[,a]))
  wilcox_test=wilcox.test(a~Risk, data=df)

  if(wilcox_test$p.value<0.001){
    myviolin=ggviolin(df, x="Risk", y="a", fill = "Risk",
             palette = c('green','red'),
             add = "boxplot",
             add.params = list(fill="white"),
             order=mygroup)+
```

```
      stat_compare_means(comparisons = mycomlist,label="p.signif")
    pdf(file=paste0(a, ".pdf"))
    print(myviolin)
    dev.off()
  }
}
```

```
####å…³æ³¨å¾®ä¿¡å…¬ä¼—å• ·ç”Ÿä¿¡ç‹‚äººå›¢é˜?
###é•‡å ˆ°ä»£ç •æŠ¥é”™ç–‰ä¸•æ‡‚çš„é—
®é¢˜å•¯ä»¥æ·»åŠ å¾®ä¿¡scikuangrenè¿›è¡Œç–”ç–
‛ ###ä½œè€…é‚®ç®±ï¼šsxkrteam@shengxinkuangren.com

#install.packages("survminer")
library(survival)
library(survminer)
setwd("C:\\Users\\qqm\\Desktop\\chromatin\\13_step13")
mydata1=read.table("Riskscore.txt",   header=T,   sep="\t",
check.names=F)
mydata2=read.table("clinical.txt",    header=T,    sep="\t",
check.names=F)
mydata3=merge(mydata1,mydata2,by="id")

head(mydata3)

clinical1="M"
clinical2="M1"
mytitle=paste0(clinical2," ","Risk")
surdata=mydata3[mydata3[,clinical1]==clinical2,]
mydiff=survdiff(Surv(survival_time, status) ~Riskgroup,data
= surdata)
myfit <- survfit(Surv(survival_time, status) ~Riskgroup,
data = surdata)
p=1-pchisq(mydiff$chisq,df=1)
if(p<0.001){
  p="p<0.001"
}else{ p=paste0("p=",sprintf("%.03f",p
  ))
}

pdf(file=paste0("survival.",clinical1,"2","_",".pdf"),
onefile = F,8,6)
ggsurvplot(myfit,
           data=surdata,
           conf.int=F,
           pval=p,
           pval.size=6,
           legend.title=mytitle,
           legend.labs=c("High risk", "Low risk"),
           xlab="Time(years)",
           break.time.by = 1,
           palette=c("red", "blue"),
```

```
          risk.table=F,
)
dev.off()

####å…³æ³¨å¾®ä¿¡å…¬ä¼—å• ·ç”Ÿä¿¡ç‹‚äººå›¢é˜?
###é•‡å ˆ°ä»£ç •æŠ¥é”™ç–‰ä¸•æ‡,çš„é—
®é¢˜å•¯ä»¥æ·»åŠ å¾®ä¿¡scikuangrenè¿›è¡Œç–”ç–
` ###ä½œè€…é‚®ç®±ï¼šsxkrteam@shengxinkuangren.com
```

```
####鍏虫敞寰俊鍏叧紬鍙风敓淇＄嫔浜哄洟闃?
###閬囦埌浠ｇ爜鎶ラ敊绛変笉鎳傜殑闂锛  鍙  互娣诲姞寰  俊 scikuangren
杩涜  绛旂枒
###浣滆€呴偖绠憋細 sxkrteam@shengxinkuangren.com



#install packages
if (!requireNamespace("BiocManager", quietly = TRUE))
  install.packages("BiocManager")
BiocManager::install("org.Hs.eg.db")

if (!requireNamespace("BiocManager", quietly = TRUE))
  install.packages("BiocManager")

BiocManager::install("clusterProfiler")


if (!requireNamespace("BiocManager", quietly = TRUE))
  install.packages("BiocManager")
BiocManager::install("enrichplot")

#library r packages
library(org.Hs.eg.db)
library(clusterProfiler)
library(enrichplot)
library(ggplot2)


setwd("C:\\Users\\qqm\\Desktop\\chromatin\\15_step15")
gene_symbol=read.table("diffgene.txt",sep="\t",check.names=F,header=T)
gene_name=as.vector(gene_symbol[,1])
id=as.data.frame(gene_name)
geneID <- mget(gene_name, org.Hs.egSYMBOL2EG, ifnotfound=NA)
geneID <- as.character(geneID)
data=cbind(gene_symbol,entrezID=geneID)
write.table(id,"id.txt",sep="\t",quote = F,row.names = F,col.names = F)
write.table(data,"name_id.txt",sep="\t",quote = F,row.names = F)

#GO
go <- enrichGO(gene=data$entrezID,
               OrgDb = org.Hs.eg.db, ont='ALL',pvalueCutoff = 0.05)#MF
CC BP
write.csv(go,"go.csv",row.names =F)
pdf("GO1.pdf",12,10)
```

```
barplot(go, drop = T, showCategory =5,split="ONTOLOGY") + facet_grid(ONTOLOGY~., scale='free')
dev.off()
pdf("GO2.pdf",12,10)
dotplot(go,showCategory = 5,split="ONTOLOGY")+ facet_grid(ONTOLOGY~., scale='free')
dev.off()
####鍏虫敞寰俊鍏絺紬鍙风敓淇＄嫃浜哄洟闃?
###閬囧埌浠ｇ爜鎶ラ敊绛変笉鐞嗚В鐨勯棶棰 鍙 互娣诲姞寰 俊 scikuangren 杩涚 绛旂枒
###浣滆€呴偖绠憋細 sxkrteam@shengxinkuangren.com

##KEGG


R.utils::setOption("clusterProfiler.download.method",'auto')
kegg <- enrichKEGG(gene = data$entrezID,organism ="human",pvalueCutoff = 0.05)
write.csv(kegg,"KEGG.csv",row.names =F)
pdf("kegg1.pdf",10,8)
barplot(kegg, showCategory =15)
dev.off()

pdf("kegg2.pdf",10,8)
dotplot(kegg,showCategory = 15)
dev.off()
####鍏虫敞寰俊鍏絺紬鍙风敓淇＄嫃浜哄洟闃?
###閬囧埌浠ｇ爜鎶ラ敊绛変笉鐞嗚В鐨勯棶棰 鍙 互娣诲姞寰 俊 scikuangren 杩涚 绛旂枒
###浣滆€呴偖绠憋細 sxkrteam@shengxinkuangren.com
```

```
####鍏虫敞寰俊鍏紒鍙风敓淇＄鎷浜哄洟闃?
###闃囧埌浠ｇ爜鎶ラ敊缁变笉鐭ユ€庢牱鏉ヨВ鍐冲彲娣诲姞寰俊 scikuangren
杩涘 缁旂粶
###浣滆€呴偖绠憋細 sxkrteam@shengxinkuangren.com


setwd("C:\\Users\\qqm\\Desktop\\chromatin\\12_step2")
data1=read.table("Riskscore.txt",header = T,sep = "\t",check.names = F)
data2=read.table("clinical.txt",header = T,sep = "\t",check.names = F)
data3=merge(data1,data2,by="id")

for (a in
colnames(data3[,3:ncol(data3)])){ scorename="Risksc
ore"
clinical=a
riskscore=data3
head(riskscore)
riskscore=riskscore[,c("id",clinical,scorename)]
colnames(riskscore)=c("id","clinical","score")

xlabel=vector()
tab1=table(riskscore[,"clinical"])
labn=length(tab1)
for(i in
  1:labn ){ xlabel=c(xlabel,names(tab1[i]))
}
mytest<-wilcox.test(score ~ clinical, data = riskscore)
p=mytest$p.value
if(p<0.001){ p="p<0
  .001"
}else{
  p=paste0("p=",sprintf("%.03f",p))
}
```


####鍏虫敞寰俊鍏紒鍙风敓淇＄鎷浜哄洟闃?
###闃囧埌浠ｇ爜鎶ラ敊缁变笉鐭ユ€庢牱鏉ヨВ鍐冲彲娣诲姞寰俊 scikuangren
杩涘 缁旂粶
###浣滆€呴偖绠憋細 sxkrteam@shengxinkuangren.com


```
mybox = boxplot(score ~ clinical, data = riskscore,outline = F, plot=F)
ymin=min(mybox$stats)
ymax = max(mybox$stats/5+mybox$stats)
y1 = max(mybox$stats/10+mybox$stats)
y12 = max(mybox$stats/12+mybox$stats)
n = ncol(mybox$stats)
```

```
pdffile=paste(clinical,".pdf",sep="")
pdf(file=pdffile,8,8)
par(mar = c(4,7,3,3))
boxplot(score ~ clinical, data = riskscore,names=xlabel,xlab = "",main=clinical,
        ylab = paste(scorename),col=c("red","green"),
        cex.main=1.6, cex.lab=1.4, cex.axis=1.3,ylim=c(ymin,ymax),outline = F)
segments(1,y1, n,y1);
segments(1,y1, 1,y12)
segments(n,y1, n,y12)
text((1+n)/2,y1,labels=p,cex=1.5,pos=3)
dev.off()
}
####鍏虫敞寰俊鍏紭細鍙风敓淇＄嫄浜哄湈闃?
###閬囧埌浠ｇ爜鎶ラ敊绛変笉鎳傜殑闂蹇   鍙  互娣诲姞寰   俊 scikuangren 杩涜  绛旂枒
###浣滆€呴偖绠憋細 sxkrteam@shengxinkuangren.com
```

```
####鍏虫敞寰俊鍏紬鍙风敓淇＄嫍浜哄湭闃?
###閬囧埌浠ｇ爜鎶ラ敊绛変笉鎳傜殑闂锛屾 鍙 互娣诲姞寰 俊 scikuangren
杩涜 绛旂枒
###浣滆€呴偖绠憋細 sxkrteam@shengxinkuangren.com


setwd("C:\\Users\\qqm\\Desktop\\chromatin\\19_step19")
library(limma)
library(pheatmap)
tcga<-read.table("All_infiltration_estimation.csv",header = T,sep =
",",check.names = F)
tcga=as.matrix(tcga)
rownames(tcga)=tcga[,1]
GeneExp=tcga[,2:ncol(tcga)]
TCGA=matrix(as.numeric(as.matrix(GeneExp)),nrow=nrow(GeneExp),dimnames
=list(rownames(GeneExp),colnames(GeneExp)))
nc=substr(rownames(TCGA),1,12)
rownames(TCGA)=nc
TCGA=avereps(TCGA)
tumor=cbind(id=row.names(TCGA),TCGA)
risk=read.table("Riskscore.txt",header = T,sep = "\t",check.names = F)
risk_mRNA=merge(risk,tumor,by="id")
rownames(risk_mRNA)=risk_mRNA$id
risk_mRNA=risk_mRNA[,-1]
myresult=data.frame()
mygroup=c("Riskscore")
for(i in
    colnames(risk_mRNA)[2:ncol(risk_mRNA)]){ mytest=wilcox.test(as.nume
    ric(risk_mRNA[,i]) ~
risk_mRNA[,"Riskscore"])
    p=mytest$p.value
  if(p<0.05){
    myresult=rbind(myresult,cbind(id=i, p))
    mygroup=c(mygroup, i)
  }
}
write.table(myresult,"myresult.txt" ,sep="\t", quote=F, row.names=F)

ht=risk_mRNA[,mygroup]

ht=ht[order(ht[,"Riskscore"]),]
cann=ht[,1,drop=F]
cann[,"Riskscore"]=factor(cann[,"Riskscore"],
unique(cann[,"Riskscore"]))
ht=t(ht[,(2:ncol(ht))])
####鍏虫敞寰俊鍏紬鍙风敓淇＄嫍浜哄湭闃?
```

###閬囧埌浠ｇ爜鎶ラ敊绛変笉鐞嗚В鐨勯棶棰　鍙　互娣诲姞寰　俊 scikuangren 杩涜 绛旂枒

###浣滆€呴偖绠憋細 sxkrteam@shengxinkuangren.com

```
rann=sapply(strsplit(rownames(ht),"_"), '[', 2)
rann=as.data.frame(rann)
row.names(rann)=row.names(ht)
colnames(rann)=c("Methods")
rann[,"Methods"]=factor(rann[,"Methods"], unique(rann[,"Methods"]))
cgap=as.vector(cumsum(table(cann[,"Riskscore"])))
rgap=as.vector(cumsum(table(rann[,"Methods"])))

mycolor=rainbow(9)
mycolor=mycolor[1:length(unique(cann[,"Riskscore"]))]
Riskscore=mycolor
names(Riskscore)=levels(factor(cann[,"Riskscore"]))
colorslist=list(Riskscore=Riskscore)

ht=matrix(as.numeric(as.matrix(ht)),nrow=nrow(ht),
          dimnames=list(rownames(ht),colnames(ht)))

pdf("immheatmap.pdf", 10,8)
pheatmap(ht,
         annotation=cann,
         annotation_row=rann,
         annotation_colors = colorslist,
         color = colorRampPalette(c(rep("blue",5), "white", rep("red",5)))(100),
         cluster_cols =F,
         cluster_rows =F,
         gaps_row=rgap,
         gaps_col=cgap,
         scale="row",
         show_colnames=F,
         show_rownames=T,
         fontsize=6,
         fontsize_row=5,
         fontsize_col=6)
dev.off()
```

####鍏虫敞寰俊鍏紬鍙凤紝姣忓ぉ$鍋浜哄洖澶?

###閬囧埌浠ｇ爜鎶ラ敊绛変笉鐞嗚В鐨勯棶棰　鍙　互娣诲姞寰　俊 scikuangren 杩涜 绛旂枒

###浣滆€呴偖绠憋細 sxkrteam@shengxinkuangren.com

```
if (!requireNamespace("BiocManager", quietly = TRUE))
    install.packages("BiocManager")
BiocManager::install(c("car", "ridge", "preprocessCore", "genefilter", "sva"))

install.packages("ggpubr")


####鍏虫敞寰俊鍏紬鍙风敓淇＄姗浜哄洟闃?
###闅囧埌浠ｇ爜鎶ラ敊缁变笉鍥傜殑闂鍙  鍙 互娣诲姞寰 俊 scikuangren 杩涚 缇旂枒
###浣滆€呴偖绠憋細 sxkrteam@shengxinkuangren.com
library(limma)
library(ggpubr)
library(pRRophetic)
library(ggplot2)
set.seed(666)



setwd("C:\\Users\\qqm\\Desktop\\chromatin\\22_step22")
mydrugs=c("A.443654", "A.770041", "ABT.263", "ABT.888", "AG.014699", "AICAR", "AKT.inhibitor.VIII", "AMG.706", "AP.24534", "AS601245", "ATRA", "AUY922", "Axitinib", "AZ628", "AZD.0530", "AZD.2281", "AZD6244", "AZD6482", "AZD7762", "AZD8055", "BAY.61.3606", "Bexarotene", "BI.2536", "BIBW2992", "Bicalutamide", "BI.D1870", "BIRB.0796", "Bleomycin", "BMS.509744", "BMS.536924", "BMS.708163", "BMS.754807", "Bortezomib", "Bosutinib", "Bryostatin.1", "BX.795", "Camptothecin", "CCT007093", "CCT018159", "CEP.701", "CGP.082996", "CGP.60474", "CHIR.99021", "CI.1040", "Cisplatin", "CMK", "Cyclopamine", "Cytarabine", "Dasatinib", "DMOG", "Docetaxel", "Doxorubicin", "EHT.1864", "Elesclomol", "Embelin", "Epothilone.B", "Erlotinib", "Etoposide", "FH535", "FTI.277", "GDC.0449", "GDC0941", "Gefitinib", "Gemcitabine", "GNF.2", "GSK269962A", "GSK.650394", "GW.441756", "GW843682X", "Imatinib", "IPA.3", "JNJ.26854165", "JNK.9L", "JNK.Inhibitor.VIII", "JW.7.52.1", "KIN001.135", "KU.55933", "Lapatinib", "Lenalidomide", "LFM.A13", "Metformin", "Methotrexate", "MG.132", "Midostaurin", "Mitomycin.C", "MK.2206", "MS.275", "Nilotinib", "NSC.87877", "NU.7441", "Nutlin.3a", "NVP.BEZ235", "NVP.TAE684", "Obatoclax.Mesylate", "OSI.906", "PAC.1", "Paclitaxel", "Parthenolide", "Pazopanib", "PD.0325901", "PD.0332991", "PD.173074", "PF.02341066", "PF.4708671", "PF.562271", "PHA.665752", "PLX4720", "Pyrimethamine", "QS11", "Rapamycin", "RDEA119", "RO.3306", "Roscovitine", "Salubrinal", "SB.216763", "SB590885", "Shikonin", "SL.0101.1", "Sorafenib", "S.Trityl.L.cysteine", "Sunitinib",
```

```
"Temsirolimus", "Thapsigargin", "Tipifarnib", "TW.37", "Vinblastine",
"Vinorelbine", "Vorinostat", "VX.680", "VX.702", "WH.4.023",
"WO2009093972", "WZ.1.84", "X17.AAG", "X681640", "XMD8.85",
"Z.LLNle.CHO", "ZM.447439")
mydrugs=c("Cyclopamine", "Cytarabine", "Dasatinib", "DMOG", "Docetaxel")
risk=read.table("Riskscore.txt",header = T,sep = "\t",check.names =
F,row.names = 1)
mRNA=read.table("tumor.txt",header = T,sep = "\t",check.names =F)
mRNA=as.matrix(mRNA)
rownames(mRNA)=mRNA[,1]
Geneexp=mRNA[,2:ncol(mRNA)]
newdf=matrix(as.numeric(as.matrix(Geneexp)),nrow=nrow(Geneexp),dimname
s=list(rownames(Geneexp),colnames(Geneexp)))
newdf=avereps(newdf)
newdf=t(newdf)

####鍏虫敞寰俊鍏綔紬鍙风敓淇＄嫔浜哄洟闃?
###闃囧埌浠ｇ爜鎶ラ敊绛变笉鍥傜殑闂蜷　鍙　互娣诲姞寰　俊 scikuangren
杩涜 绛旂枒
###浣滆€呴偖绠憋細 sxkrteam@shengxinkuangren.com
for (drugi in mydrugs){
drug_sen=pRRopheticPredict(newdf, drugi, selection=1)
drug_sen=drug_sen[drug_sen!="NaN"]
coid=intersect(row.names(risk), names(drug_sen))
risk=risk[coid, "Risk",drop=F]

drug_sen=drug_sen[coid]
drugrisk=cbind(risk, drug_sen)
risk$Risk=factor(risk$Risk, levels=c("Low", "High"))
mygroup=levels(factor(risk[,"Risk"]))
mycom=combn(mygroup, 2)
mycomlist=list()
for(b in 1:ncol(mycom)){mycomlist[[b]]<-mycom[,b]}
wilcox_test=wilcox.test(drug_sen~Risk, drugrisk)


if (wilcox_test$p.value<0.05){
myboxplot=ggboxplot(drugrisk, x="Risk", y="drug_sen", fill = "Risk",
palette = c('green','red'),ylab=paste0(drugi, " senstivity (IC50)"),
                   add.params = list(fill="white"),
                   order=mygroup)+
    stat_compare_means(comparisons = mycomlist)
  pdf(file=paste0(drugi, ".pdf"))
  print(myboxplot)
  dev.off()
```

}
}

####鍏虫敞寰俊鍏紼紬鍙风敓淇＄嫄浜哄洟闃?
###閬囧埌浠ｇ爜鎶ラ敊绛変笉鍥偣殑闂蹉　鍙　互娣诲姞寰　绋俊 scikuangren
杩涜　绛旂枒
###浣滆€呴偖绠憋細 sxkrteam@shengxinkuangren.com

```
####鍏虫敞寰俊鍏紬鍙风敓淇＄媿浜哄湪闃?
###闃囧埌浠ｇ爜鎶ラ敊绛変笉鎳傜殑闂鐩 鍙 互娣诲姞寰 俊 scikuangren
杩涜 绛旂枒
###浣滆€呴偖绠憋細 sxkrteam@shengxinkuangren.com


library(pheatmap)
setwd("C:\\Users\\qqm\\Desktop\\chromatin\\11_step11")
data1=read.table("Riskscore.txt",header = T,sep = "\t",check.names = F)
data2=read.table("clinical.txt",header = T,sep = "\t",check.names = F)
data3=merge(data1,data2,by="id")
write.table(data3,"clinicalheat.txt",sep = "\t",quote = F,row.names =
F)



####鍏虫敞寰俊鍏紬鍙风敓淇＄媿浜哄湪闃?
###闃囧埌浠ｇ爜鎶ラ敊绛変笉鎳傜殑闂鐩 鍙 互娣诲姞寰 俊 scikuangren
杩涜 绛旂枒
###浣滆€呴偖绠憋細 sxkrteam@shengxinkuangren.com

ht=read.table("geneheat.txt",sep="\t",header=T,
              row.names=1,check.names=F)

ht=t(ht)

Group=read.table("clinicalheat.txt",sep="\t",
                 header=T,row.names=1,check.names=F)
Group=Group[order(Group$Riskscore),]
ht=ht[,row.names(Group)]

pdf("heatmap.pdf",10,8)
pheatmap(ht, annotation=Group,
         color = colorRampPalette(c(rep("blue",3), "white",
rep("red",3)))(100),
         cluster_cols =F,
         fontsize=7,
         fontsize_row=8,
         scale="row",
         show_colnames=F,
         fontsize_col=3)
dev.off()

####鍏虫敞寰俊鍏紬鍙风敓淇＄媿浜哄湪闃?
###闃囧埌浠ｇ爜鎶ラ敊绛変笉鎳傜殑闂鐩 鍙 互娣诲姞寰 俊 scikuangren
杩涜 绛旂枒
```

###涴滆€呴偖绠憋細 sxkrteam@shengxinkuangren.com

####å…³æ³¨å¾®ä¿¡å…¬ä¼—å• ·ç”Ÿä¿¡ç‹‚ä°°å›¢é˜?
###é•‡å˜°ä»£ç •æŠ¥é”™ç–‰ä¸•æ‡‚çš„é—
®é¢˜å•¯ä»¥æ·»åŠ å¾®ä¿¡scikuangrenè¿›è¡Œç–”ç–
‵ ###ä½œè€…é‚®ç®±ï¼šsxkrteam@shengxinkuangren.com

```
library(pheatmap)
setwd("C:\\Users\\qqm\\Desktop\\chromatin\\8_step8")
riskdata=read.table("Riskscore.txt",header = T,sep = "\t",row.names = 1)
riskdata=riskdata[order(riskdata$Riskscore),]
nriskdata=riskdata[c(3:(ncol(riskdata)-2))]
nriskdata=t(nriskdata)
nriskdata=log2(nriskdata+1)
mycolor=list()
mycolor2=c("green", "red")
names(mycolor2)=c("Low", "High")
mycolor[["Riskgroup"]]=mycolor2
ann=data.frame(Riskgroup=riskdata[,ncol(riskdata)])
rownames(ann)=rownames(riskdata)


pdf("heatmap.pdf",10,8)
pheatmap(nriskdata,
        annotation=ann,
        annotation_colors = mycolor,
        cluster_cols = F,
        cluster_rows = F,
        show_colnames = F,
        scale="row",
        color= colorRampPalette(c("green", "black", "red"))(50),
        fontsize_col=4,
        fontsize=8,
        fontsize_row=8)
dev.off()
```

####å…³æ³¨å¾®ä¿¡å…¬ä¼—å• ·ç”Ÿä¿¡ç‹‚ä°°å›¢é˜?
###é•‡å˜°ä»£ç •æŠ¥é”™ç–‰ä¸•æ‡‚çš„é—
®é¢˜å•¯ä»¥æ·»åŠ å¾®ä¿¡scikuangrenè¿›è¡Œç–”ç–
‵ ###ä½œè€…é‚®ç®±ï¼šsxkrteam@shengxinkuangren.com

```
####鍏虫敞寰俊鍏綊紬鍙风敓淇＄鐑浜哄洟闃?
###闅囧埌浠ｇ爜鎶ラ敊绛変笉鎳傜殑闂鏌   鍙  互娣诲姞寰   绨俊 scikuangren
杩涜 绛旂枒
###浣滆€呴偖绠憋細 sxkrteam@shengxinkuangren.com

setwd("C:\\Users\\77632\\Desktop\\chromatin\\step7")
library(timeROC)
library(survival)
TCGA<-read.table("Riskscore.txt",header=T,sep="\t")
predict_1_year<- 1
predict_3_year<- 3
predict_5_year<- 5

ROC<-timeROC(T=TCGA$survival_time,delta=TCGA$status,
             marker=TCGA$Riskscore,cause=1,
             weighting="marginal",


times=c(predict_1_year,predict_3_year,predict_5_year),ROC=T)

pdf("ROC.pdf")
plot(ROC,time=predict_1_year,title=F,lwd=3)
plot(ROC,time=predict_3_year,col="yellow",add=T,title=F,lwd=3)
plot(ROC,time=predict_5_year,col="blue",add=T,title=F,lwd=3)
legend("bottomright",
       c(paste("AUC of 1 year survival: ",round(ROC$AUC[1],3)),
         paste("AUC of 3 year survival: ",round(ROC$AUC[2],3)),
         paste("AUC                of                5                year
surviva",round(ROC$AUC[3],3))),col=c("red","yellow","blue"),lwd=3)
dev.off()
```

####鍏虫敞寰俊鍏紬鍙风敓淇＄嫔浜哄洟闃?
###閬囧埌浠ｇ爜鎶ラ敊绛変竴鍒囬棶棰樸   鍙 互娣诲姞寰   俊 scikuangren
杩涚 绛旂枒
###浣滆€呴偖绠憋細 sxkrteam@shengxinkuangren.com

```
library(pheatmap)
setwd("C:\\Users\\qqm\\Desktop\\chromatin\\3_step3")
ht=read.table("diffgeneEXP.txt",header = T,sep = "\t",row.names = 1,check.names = F)
ht=log2(ht+1)
Group=c(rep("N",72),rep("T",536))
names(Group)=colnames(ht)
Group=as.data.frame(Group)

ht=ht[1:50,]

pdf("heatmap.pdf",10,8)
pheatmap(ht, annotation=Group,
         color = colorRampPalette(c("blue", "white", "red"))(150),
         cluster_cols =F,show_colnames = F)
dev.off()
```

####鍏虫敞寰俊鍏紬鍙风敓淇＄嫔浜哄洟闃?
###閬囧埌浠ｇ爜鎶ラ敊绛変竴鍒囬棶棰樸   鍙 互娣诲姞寰   俊 scikuangren
杩涚 绛旂枒
###浣滆€呴偖绠憋細 sxkrteam@shengxinkuangren.com

```
####鍏虫敞

library(survival)
setwd("C:\\Users\\qqm\\Desktop\\chromatin\\4_step4")
dt1=read.table("diffgeneEXP.txt",header = T,sep = "\t",check.names = F)
st<- which(substr(colnames(dt1),14,15) == '11')
tumor=dt1[,-st]
nc=substr(colnames(tumor),1,12)
colnames(tumor)=nc
tumor=t(tumor)
write.table(tumor,"tumor.txt",sep = "\t",quote = F,col.names = F)
dt4=read.table("tumor.txt",header = T,sep = "\t",check.names =F)
clidata=read.table("time.txt",header = T,sep = "\t",check.names =F)

cliexp=merge(clidata,dt4,by="id")
write.table(cliexp,"timeexp.txt",sep = "\t",quote = F,row.names = F)

inputfile="timeexp.txt"
data1=read.table(inputfile,header = T,sep = "\t",check.names =F,row.names = 1)
data1$survival_time=data1$survival_time/365

coxf<-function(x){
  fmla1 <- as.formula(Surv(survival_time,status)~data1[,x])
  mycox <- coxph(fmla1,data=data1)
}

newdf=data.frame()
for(a in
  colnames(data1[,3:ncol(data1)])){ mycox=coxf(a)
  coxResult = summary(mycox)
  newdf=rbind(newdf,
              cbind(id=a,
                    HR=coxResult$conf.int[,"exp(coef)"],
                    HR_95L=coxResult$conf.int[,"lower .95"],
                    HR_95U=coxResult$conf.int[,"upper .95"],
                    P=coxResult$coefficients[,"Pr(>|z|)"]
              ))

}
newdf=newdf[(newdf$P<0.01),]
write.table(newdf,"result.txt",sep="\t",row.names=F,quote=F)

###閬囧埌浠ｇ爜鎶ラ敊绛変笉鐭ヤ簬鎬庝箞瑙ｅ喅鐨勯   鍙    互娣诲姞寰 俊 scikuangren
```

```
杩涜  绛旂枒
###浣滆€呴偖绠憋細 sxkrteam@shengxinkuangren.com



###################forest#################
####鍏虫敞寰俊鍏紬鍙凤紝鍏嶈垂瀛︿範鐢熶俊$鍒嗘瀽?
###閬囧埌涓ｇ爜鍗ラ敊绛変笉鐭ョ殑闂蹇   鍙  互娣诲姞寰   俊 scikuangren
杩涜 绛旂枒
###浣滆€呴偖绠憋細 sxkrteam@shengxinkuangren.com



HRdf1<- read.table("result.txt", header=T, sep="\t", check.names=F,
row.names=1)
idname <- rownames(HRdf1)
HR=sprintf("%.4f",HRdf1[,"HR"])
HR_95L=sprintf("%.4f",HRdf1[,"HR_95L"])
HR_95U=sprintf("%.4f",HRdf1[,"HR_95U"])
P=HRdf1[,"P"]
P=ifelse(P<0.001, "<0.001", sprintf("%.4f", P))

pdf("forest.pdf")
snum <- nrow(HRdf1)
snum2 <- snum+1
layout(matrix(c(1,2),nc=2),width=c(3,2.5))
par(mar=c(4,2.5,2,1))
plot(1,xlim= c(0,3),ylim=c(1,snum2),type="n",axes=F,xlab="",ylab="")
text(0,snum:1,idname,adj=0,cex=0.8)
text(1.5-0.5*0.2,snum:1,P,adj=1,cex=0.8);text(1.5-
0.5*0.2,snum+1,'pvalue',cex=0.8,font=2,adj=1)
text(3.1,snum:1,                                    paste0(HR,"(",HR_95L,"-
",HR_95U,")"),adj=1,cex=0.8);text(3.1,snum+1,'Hazard
ratio',cex=0.8,font=2,adj=1)
par(mar=c(4,1,2,1),mgp=c(2,0.5,0))
xlim = c(0,max(as.numeric(HR_95L),as.numeric(HR_95U)))
plot(1,xlim=c(0,3),ylim=c(1,snum2),type="n",axes=F,ylab="",xaxs="i",xl
ab="Hazard ratio")
arrows(as.numeric(HR_95L),snum:1,as.numeric(HR_95U),
        snum:1,angle=90,code=3,length=0.05,col="skyblue",lwd=2.5)
abline(v=1,col="black",lty=2,lwd=2)
points(as.numeric(HR), snum:1, pch = 15,
        col = ifelse(as.numeric(HR) > 1, "red", "green")
        , cex=1.5)
axis(1)
```

```
dev.off()

########################################
####鍏虫敞寰俊鍏絺紬鍙风敓淇＄嬀浜哄洟闃?
###閬囧埌浠ｇ爜鎶ラ敊绛変笉鎳傜殑闂鐩   鍙  互娣诲姞寰   绱俊 scikuangren
杩涜 绛旂枒
###浣滆€呴偖绠憋細 sxkrteam@shengxinkuangren.com
```

```
######################################
####鍏虫敞寰俊鍏紬鍙风敓淇＄嫔浜哄洟闃?
###閬囧埌浠ｇ爜鍋ラ敊绛変笉鎳傜殑闂蝘  鍙  互娣诲姞寰  俊 scikuangren
杩涜  绛旀枒
###浣滆€呴偖绠憋細 sxkrteam@shengxinkuangren.com


library(survival)
setwd("C:\\Users\\qqm\\Desktop\\chromatin\\9_step9")


data1=read.table("Riskscore.txt",header = T,sep = "\t",check.names = F)
data2<-read.table("clinical.txt",header=T,sep="\t",check.names = F)
data3=merge(data1,data2,by="id")
write.table(data3,"cr.txt",quote = F,sep = "\t",row.names = F)



#############################    鍗    曞    彔    绡    湯
ox############################################
ndata1=read.table("cr.txt",header    =    T,sep    =    "\t",check.names
=F,row.names = 1)
coxf<-function(x){
  fmla1 <- as.formula(Surv(survival_time,status)~ndata1[,x])
  mycox <- coxph(fmla1,data=ndata1)
}

newdf=data.frame()
for(a in
  colnames(ndata1[,3:ncol(ndata1)])){ mycox=coxf(a)
  coxResult = summary(mycox)
  newdf=rbind(newdf,
              cbind(id=a,
                    HR=coxResult$conf.int[,"exp(coef)"],
                    HR_95L=coxResult$conf.int[,"lower .95"],
                    HR_95U=coxResult$conf.int[,"upper .95"],
                    P=coxResult$coefficients[,"Pr(>|z|)"]
              ))

}
write.table(newdf,"result.txt",sep="\t",row.names=F,quote=F)

################################forest################################
#############
HRdf1=read.table("result.txt",header=T,sep="\t",row.names=1,check.name
```

```
s=F)
idname <- rownames(HRdf1)
HR=sprintf("%.4f",HRdf1[,"HR"])
HR_95L=sprintf("%.4f",HRdf1[,"HR_95L"])
HR_95U=sprintf("%.4f",HRdf1[,"HR_95U"])
P=HRdf1[,"P"]
P=ifelse(P<0.001, "<0.001", sprintf("%.4f", P))

pdf("forest1.pdf",8,6)
snum <- nrow(HRdf1)
snum2 <- snum+1
layout(matrix(c(1,2),nc=2),width=c(3,2.5))
par(mar=c(4,2.5,2,1))
plot(1,xlim= c(0,3),ylim=c(1,snum2),type="n",axes=F,xlab="",ylab="")
text(0,snum:1,idname,adj=0,cex=0.8)
text(1.5-0.5*0.2,snum:1,P,adj=1,cex=0.8);text(1.5-
0.5*0.2,snum+1,'pvalue',cex=0.8,font=2,adj=1)
text(3.1,snum:1,                                   paste0(HR,"(",HR_95L,"-
",HR_95U,")"),adj=1,cex=0.8);text(3.1,snum+1,'Hazard
ratio',cex=0.8,font=2,adj=1)
par(mar=c(4,1,2,1),mgp=c(2,0.5,0))
xlim = c(0,max(as.numeric(HR_95L),as.numeric(HR_95U)))
plot(1,xlim=c(0,7),ylim=c(1,snum2),type="n",axes=F,ylab="",xaxs="i",xl
ab="Hazard ratio")###xlim=c(0,7)中 7 可以调节Hazard ratio 宽度
arrows(as.numeric(HR_95L),snum:1,as.numeric(HR_95U),
        snum:1,angle=90,code=3,length=0.05,col="skyblue",lwd=2.5)
abline(v=1,col="black",lty=2,lwd=2)
points(as.numeric(HR), snum:1, pch = 15,
        col ="green"
        , cex=1.5)
axis(1)
dev.off()

#########################################
####鍏虫敞寰俊鍏綘紬鍙风敓淇＄嫄浜哄洟闃?
###閬囧埌浠ｇ爜鎶ラ敊绛変竴鍥偓殑闂蹇    鍙  互娣诲姞寰    俊 scikuangren
杩涜  绛旂枒
###涜濡€呴偖绠憋細 sxkrteam@shengxinkuangren.com



###############################################     澶    氬    粂    绡    湀
ox#######################################
fmla2 <- as.formula(Surv(survival_time,status)~.)
mycox2 <- coxph(fmla2,data=ndata1)
```

```
coxResult2=summary(mycox2)
newdf2=cbind(
  HR=coxResult2$conf.int[,"exp(coef)"],
  HR_95L=coxResult2$conf.int[,"lower .95"],
  HR_95U=coxResult2$conf.int[,"upper .95"],
  P=coxResult2$coefficients[,"Pr(>|z|)"])
newdf2=cbind(id=row.names(newdf2),newdf2)

write.table(newdf2,"result2.txt",sep="\t",row.names=F,quote=F)

##################forest#################################################
################
HRdf1=read.table("result2.txt",header=T,sep="\t",row.names=1,check.nam
es=F)
idname <- rownames(HRdf1)
HR=sprintf("%.4f",HRdf1[,"HR"])
HR_95L=sprintf("%.4f",HRdf1[,"HR_95L"])
HR_95U=sprintf("%.4f",HRdf1[,"HR_95U"])
P=HRdf1[,"P"]
P=ifelse(P<0.001, "<0.001", sprintf("%.4f", P))

pdf("forest2.pdf",8,6)
snum <- nrow(HRdf1)
snum2 <- snum+1
layout(matrix(c(1,2),nc=2),width=c(3,2.5))
par(mar=c(4,2.5,2,1))
plot(1,xlim= c(0,3),ylim=c(1,snum2),type="n",axes=F,xlab="",ylab="")
text(0,snum:1,idname,adj=0,cex=0.8)
text(1.5-0.5*0.2,snum:1,P,adj=1,cex=0.8);text(1.5-
0.5*0.2,snum+1,'pvalue',cex=0.8,font=2,adj=1)
text(3.1,snum:1,                                    paste0(HR,"(",HR_95L,"-
",HR_95U,")"),adj=1,cex=0.8);text(3.1,snum+1,'Hazard
ratio',cex=0.8,font=2,adj=1)
par(mar=c(4,1,2,1),mgp=c(2,0.5,0))
xlim = c(0,max(as.numeric(HR_95L),as.numeric(HR_95U)))
plot(1,xlim=c(0,5),ylim=c(1,snum2),type="n",axes=F,ylab="",xaxs="i",xl
ab="Hazard ratio")
arrows(as.numeric(HR_95L),snum:1,as.numeric(HR_95U),
       snum:1,angle=90,code=3,length=0.05,col="skyblue",lwd=2.5)
abline(v=1,col="black",lty=2,lwd=2)
points(as.numeric(HR), snum:1, pch = 15,
       col = "red"
       , cex=1.5)
axis(1)
```

```
dev.off()

####################################
####鍏虫敞寰俊鍏綔鍙鍙风敓淇＄娲浜哄湪闃?
###闃囧埌浠ｇ爥鎶ラ敊绛変笉鍥伭殑闂蜒    鍙  互娣诲姞寰    绻俊 scikuangren
杩涜  绛旂枒
###浣滆€呴偖绠憋細 sxkrteam@shengxinkuangren.com
```

```
####鍏虫敞寰俊鍏紬鍙风敓淇＄嬀浜哄湪闃?
###閬囧埌浠ｇ爜鎶ラ敊绛変笉鎳傜殑闂锛  鍙  互娣诲姞寰  俊 scikuangren
杩涜 绛旂枒
###浣滆€呴偖绠憋細 sxkrteam@shengxinkuangren.com

#install.packages("glmnet")
library(glmnet)
library(survival)
setwd("C:\\Users\\qqm\\Desktop\\chromatin\\5_step5")


mydata1<-read.table("timeexp.txt",header=T,sep="\t",row.names            =
1,check.names = F,stringsAsFactors = F)
mydata2=read.table("result.txt",header=T,sep="\t",check.names=F)
mydata1=mydata1[,c("survival_time","status",as.vector(mydata2[,1]))]
mydata1$survival_time=mydata1$survival_time/365

v1<-as.matrix(mydata1[,c(3:ncol(mydata1))])
v2 <- as.matrix(Surv(mydata1$survival_time,mydata1$status))

myfit <- glmnet(v1, v2, family = "cox")
pdf("lambda.pdf")
plot(myfit, xvar = "lambda", label = TRUE)
dev.off()

myfit2 <- cv.glmnet(v1, v2, family="cox",nfolds = 10)
pdf("min.pdf")
plot(myfit2)
abline(v=log(c(myfit2$lambda.min,myfit2$lambda.1se)),lty="dashed")
dev.off()
```


```
####鍏虫敞寰俊鍏紬鍙风敓淇＄嬀浜哄湪闃?
###閬囧埌浠ｇ爜鎶ラ敊绛変笉鎳傜殑闂锛  鍙  互娣诲姞寰  俊 scikuangren
杩涜 绛旂枒
###浣滆€呴偖绠憋細 sxkrteam@shengxinkuangren.com
```


```
coe <- coef(myfit, s = myfit2$lambda.min)
act_index <- which(coe != 0)
act_coe <- coe[act_index]
lassogene=row.names(coe)[act_index]
gene_coef=cbind(id=lassogene,coef=act_coe)
write.table(gene_coef,"gene_coef.txt",sep="\t",quote=F,row.names=F)
```


```
####鍏虫敞寰俊鍏紬鍙风敓淇＄嬀浜哄湪闃?
###閬囧埌浠ｇ爜鎶ラ敊绛変笉鎳傜殑闂锛  鍙   互娣诲姞寰 俊 scikuangren
```

```
杩涚   绛旀枒
###浣滆€呴偖绠憋細 sxkrteam@shengxinkuangren.com

mygeneEXP=mydata1[,lassogene]
expcoef=function(x){crossprod(as.numeric(x),act_coe)}
Riskscore=apply(mygeneEXP,1,expcoef)
Riskgroup=as.vector(ifelse(Riskscore>median(Riskscore),"High","Low"))
newdf2=cbind(mydata1[,c("survival_time","status",lassogene)],Riskscore
=as.vector(Riskscore),Riskgroup)
newdf3=cbind(id=rownames(newdf2),newdf2)
write.table(newdf3,"Riskscore.txt",sep="\t",quote=F,row.names=F)

####鍏虫敞寰俊鍏紬鍙风敓淇＄嫍浜哄湀闃?
###閬囧埌浠ｇ爜鎶ラ敊绛变笉鍒扮殑闂鏈   鍙  互娣诲姞寰   俊 scikuangren
杩涚  绛旀枒
###浣滆€呴偖绠憋細 sxkrteam@shengxinkuangren.com
```

```
####å…³æ³¨å¾®ä¿¡å…¬ä¼—å• ·ç”Ÿä¿¡ç‹,äººå›¢é˜?
###é•‡å^°ä»£ç •æŠ¥é”™ç-‰ä¸•æ‡,çš„é—
®é¢˜å•¯ä»¥æ·»åŠ å¾®ä¿¡scikuangrenè¿›è¡Œç-”ç-
‵ ###ä½œè€…é‚®ç®±ï¼šsxkrteam@shengxinkuangren.com

#install.packages("survminer")
library(survival)
library(survminer)
setwd("C:\\Users\\qqm\\Desktop\\chromatin\\6_step6")
mydata=read.table("Riskscore.txt",    header=T,    sep="\t",
check.names=F)
mydiff=survdiff(Surv(survival_time, status) ~Riskgroup,data
= mydata)
myfit <- survfit(Surv(survival_time, status) ~ Riskgroup,
data = mydata)
p=1-pchisq(mydiff$chisq,df=1)
if(p<0.001){
  p="p<0.001"
    }else{ p=paste0("p=",sprintf("%.03f
      ",p))
    }

pdf("survival_risk.pdf",onefile = FALSE,10,8)
ggsurvplot(myfit,
                   data=mydata,
                   conf.int=T,
                   pval=p,
                   pval.size=6,
                   legend.title="Risk",
                   legend.labs=c("High risk", "Low risk"),
                   xlab="Time(years)",
                   break.time.by = 1,
                   palette=c("red", "blue"),
                   risk.table=TRUE,
                   risk.table.title="",
                   risk.table.col = "strata",
                   risk.table.height=.25)
dev.off()

####å…³æ³¨å¾®ä¿¡å…¬ä¼—å• ·ç”Ÿä¿¡ç‹,äººå›¢é˜?
###é•‡å^°ä»£ç •æŠ¥é”™ç-‰ä¸•æ‡,çš„é—
®é¢˜å•¯ä»¥æ·»åŠ å¾®ä¿¡scikuangrenè¿›è¡Œç-”ç-
‵ ###ä½œè€…é‚®ç®±ï¼šsxkrteam@shengxinkuangren.com
##########################################################
```

```
###################
#######################################riskline###########
###################
myrisk=read.table("Riskscore.txt",sep="\t",
               header=T,row.names=1,check.names=F)

head(myrisk)
myrisk=myrisk[order(myrisk$Riskscore),]
mygroup=myrisk[,"Riskgroup"]
ll=length(mygroup[mygroup=="Low"])
hl=length(mygroup[mygroup=="High"])
lm=max(myrisk$Riskscore[mygroup=="Low"])
line=myrisk[,"Riskscore"]
line[line>10]=10
pdf("riskline.pdf",8,6)
plot(line, type="p", pch=16,
    xlab="Patients (increasing risk socre)", ylab="Risk
score",
    col=c(rep("green",ll),rep("red",hl)) )
abline(h=lm,v=ll,lty=2)
dev.off()



pdf("riskpoint.pdf",8,6)
plot(myrisk$survival_time, pch=16,
    xlab="Patients (increasing risk socre)", ylab="Survival
time (years)",
    col=ifelse(myrisk$status=="1","red","blue"))
legend("topright",                               c("Dead",
"Alive"),pch=16,col=c("red","blue"),cex=1.2)
abline(v=ll,lty=2)
dev.off()


####å…³æ³¨å¾®ä¿¡å…¬ä¼—å•·ç”Ÿä¿¡ç‹‚ä°ºå›¢é˜?
###é•‡å̂°ä»£ç •æŠ¥é”™ç–‰ä¸•æ‡‚çš„é—
®é¢˜å•¯ä»¥æ·»åŠ å¾®ä¿¡scikuangrenè¿›è¡Œç–”ç–
‛ ###ä½œè€…é‚®ç®±ï¼šsxkrteam@shengxinkuangren.com
```

```
####鍏虫敞寰俊鍏紬鍙鋆风敓淇＄鐙傚悍浜?
###閬囧埌涓ｇ爜鎶ラ敊绛変笉鎳傜殑闂蹇 鍙 互娣诲姞寰 俊 scikuangren
杩涜 绛旂枒
###浣滆€呴偖绠憗細 sxkrteam@shengxinkuangren.com


setwd("C:\\Users\\qqm\\Desktop\\chromatin\\10_step10")
risk=read.table("Riskscore.txt",header = T,sep = "\t")
clinical=read.table("clinical.txt",header = T,sep = "\t")
riskcli=merge(risk,clinical,by="id")
write.table(riskcli,"riskcli.txt",quote = F,sep = "\t",row.names = F)

mykf=read.table("riskcli.txt",header=T,sep="\t",check.names=F)
head(mykf)
group1="Riskgroup"
group2="age" #
kfresult=mykf[,c(group1,group2)]
mytable=table(kfresult)
chisq.test(mytable)
mytable


fisher.test(mytable)

#age  p-value  =  0.3656
#sex p-value = 0.006957
#grade p-value = 7.595e-09
#stage p-value = 1.099e-06
#pathologic_T p-value = 5.469e-06
#pathologic_M p-value = 5.149e-05
```


```
####鍏虫敞寰俊鍏紬鍙鋆风敓淇＄鐙傚悍浜?
###閬囧埌涓ｇ爜鎶ラ敊绛変笉鎳傜殑闂蹇 鍙 互娣诲姞寰 俊 scikuangren
杩涜 绛旂枒
###浣滆€呴偖绠憗細 sxkrteam@shengxinkuangren.com
```

```
####鍏虫敞寰俊鍏紭紬鍙风敓淇＄姹浜哄洟闃?
###閬囧埌浠ｇ爜鎶ラ敊绛変笉鎳傜殑闂鏃   鍙  互娣诲姞寰   缮俊 scikuangren
杩涜  绛旂枒
###浣滆€呴偖绠憋細 sxkrteam@shengxinkuangren.com


setwd("C:\\Users\\qqm\\Desktop\\chromatin\\2_step2")
library(limma)
m6Agene=read.table("BMs_gene_mRNA.txt",header        =       T,sep        =
"\t",check.names = F)
m6Agene=as.matrix(m6Agene)
rownames(m6Agene)=m6Agene[,1]
a=grep("///",m6Agene[,1])
geneE=m6Agene[,2:ncol(m6Agene)]
newgene=matrix(as.numeric(as.matrix(geneE)),nrow=nrow(geneE),
               dimnames=list(rownames(geneE),colnames(geneE)))
newgene=avereps(newgene)
newgene=newgene[rowMeans(newgene)>0,]
newdf=data.frame()

group1=72
group2=536

for(a in

row.names(newgene)){ mydata=rbind(geneexp=newgene[a,],mygroup=c(rep(1,group1)

,rep(2,group2)
))
  mydata=as.matrix(t(mydata))
  wilcoxTest<-wilcox.test(geneexp ~ mygroup, data=mydata)
  group1mea=mean(newgene[a,1:group1])
  group2mea=mean(newgene[a,(group1+1):ncol(newgene)])
  logFC=log2(group2mea)-log2(group1mea)
  p=wilcoxTest$p.value
  group1medi=median(newgene[a,1:group1])
  group2medi=median(newgene[a,(group1+1):ncol(newgene)])
  diffmedi=group2medi-group1medi
    newdf=rbind(newdf,
                cbind(gene=a,
                      group1mea=group1mea,
                      group2mea=group2mea,
                      logFC=logFC,
                      p=p))


}
```

```
p=newdf[,"p"]
FDR=p.adjust(as.numeric(as.vector(p)),method="fdr")
newdf=cbind(newdf,FDR=FDR)
newdf=na.omit(newdf)
write.table(newdf,"allgene.txt",sep="\t",row.names=F,quote=F)

####鍏虫敞寰俊鍏紸細鍙风敓淇＄嫍浜哄洟闃?
###閬囧埌浠ｇ爜鎶ラ敊绛変竴鍥氬殑闂锛  鍙  互娣诲姞寰  俊 scikuangren
杩涚  绛旂枒
###浣滆€呴偖绠憋細 sxkrteam@shengxinkuangren.com


Diffgene=newdf[(abs(as.numeric(as.vector(newdf$logFC)))>1               &
as.numeric(as.vector(newdf$FDR))<0.05),]
Diffgene=na.omit(Diffgene)
write.table(Diffgene,"diffgene.txt",sep="\t",row.names=F,quote=F)
ndf=newgene[Diffgene$gene,]
diffgeneEXP=cbind(id=row.names(ndf),ndf)
write.table(diffgeneEXP,"diffgeneEXP.txt",sep="\t",row.names=F,quote=F)
```

```
####鍏虫敞寰俊鍏紬鍙风敓淇＄嫄浜哄涔闃?
###闃 library(regplot)囧垠浠ｇ爜鎶ラ敊绛变笉鍥炲殑闂鐩 鍙 互娣诲姞寰俊 scikuangren 杩涜 绛旂枒
###浣滆€呴偖绠憋細 sxkrteam@shengxinkuangren.com

install.packages("regplot")
install.packages("rms")
library(survival)
library(regplot)
library(rms)

setwd("C:\\Users\\qqm\\Desktop\\chromatin\\14_step14")

data1=read.table("cr.txt",header = T,sep = "\t",check.names =F,row.names = 1)
#nomogram
nomcox=coxph(Surv(survival_time, status) ~ . , data = data1)
regplot(nomcox,
        plots = c("bars", "boxes"),
        clickable=F,
        title=NULL,
        points=T,
        droplines=T,
        observation=data1[9,],
        rank="sd",
        failtime = c(1,3,5),
        prfail = F)

####鍏虫敞寰俊鍏紬鍙风敓淇＄嫄浜哄涔闃?
###闃囧垠浠ｇ爜鎶ラ敊绛变笉鍥炲殑闂鐩 鍙 互娣诲姞寰俊 scikuangren 杩涜 绛旂枒
###浣滆€呴偖绠憋細 sxkrteam@shengxinkuangren.com



head(data1)
#鏍″噯鏇茬嚎
pdf("calibration.pdf",10,8)
#1 year
mx1 <- cph(Surv(survival_time, status) ~age+gender+grade+stage+pathologic_T+pathologic_M+Riskscore, x=T, y=T, surv=T, data=data1, time.inc=1)
cal1 <- calibrate(mx1, cmethod="KM", method="boot", u=1, m=(nrow(data1)/3), B=1000)
plot(cal1, xlim=c(0,1), ylim=c(0,1),
     xlab="Nomogram-predicted Overall survival (%)", ylab="Observed
```

```
Overall survival (%)", lwd=1.5, col="green", sub=F)
#3 year
mx2 <- cph(Surv(survival_time, status) ~
age+gender+grade+stage+pathologic_T+pathologic_M+Riskscore, x=T, y=T,
surv=T, data=data1, time.inc=3)
cal2 <- calibrate(mx2, cmethod="KM", method="boot", u=3,
m=(nrow(data1)/3), B=1000)
plot(cal2, xlim=c(0,1), ylim=c(0,1), xlab="", ylab="", lwd=1.5,
col="blue", sub=F, add=T)

#5 year
mx3 <- cph(Surv(survival_time, status)
~age+gender+grade+stage+pathologic_T+pathologic_M+Riskscore, x=T, y=T,
surv=T, data=data1, time.inc=5)
cal3 <- calibrate(mx3, cmethod="KM", method="boot", u=5,
m=(nrow(data1)/3), B=1000)
plot(cal3, xlim=c(0,1), ylim=c(0,1), xlab="", ylab="", lwd=1.5,
col="red", sub=F, add=T)
legend('bottomright', c('1-year', '3-year', '5-year'),
       col=c("green","blue","red"), lwd=1.5, bty = 'n')
dev.off()

####鍏虫敞寰俊鍏絺紬鍙风敓淇＄嬀浜哄湪闃?
###閬囧埌浠ｇ爜鎶ラ敊绛变笉鏁傜殑闂棦    鍙  互娣诲姞寰    绾俊 scikuangren
杩涜  绛旂枒
###浣滆€呴偖绠憋細 sxkrteam@shengxinkuangren.com
```
